# Evaluating Maturity Models in Healthcare Information Systems: A Comprehensive Review

**DOI:** 10.3390/healthcare13151847

**Published:** 2025-07-29

**Authors:** Jorge Gomes, Mário Romão

**Affiliations:** 1 ECEO, Universidade Lusófona, 1749-024 Lisboa, Portugal; 2 ADVANCE/ISEG Research, ISEG—Instituto Superior de Economia e Gestão, 1200-781 Lisboa, Portugal; mario.romao@iseg.ulisboa.pt

**Keywords:** Healthcare Information Systems (HISs), Maturity Models, Health Information Technology (HIT), digital health, interoperability, process management, HIS implementation, health system performance

## Abstract

Healthcare Information Systems (HISs) are essential for improving care quality, managing chronic diseases, and supporting clinical decision-making. Despite significant investments, HIS implementations often fail due to the complexity of healthcare environments. Maturity Models (MMs) have emerged as tools to guide organizational improvement by assessing readiness, process efficiency, technology adoption, and interoperability. This study presents a comprehensive literature review identifying 45 Maturity Models used across various healthcare domains, including telemedicine, analytics, business intelligence, and electronic medical records. These models, often based on Capability Maturity Model Integration (CMMI), vary in structure, scope, and maturity stages. The findings demonstrate that structured maturity assessments help healthcare organizations plan, implement, and optimize HIS more effectively, leading to enhanced clinical and operational performance. This review contributes to an understanding of how different MMs can support healthcare digital transformation and provides a resource for selecting appropriate models based on specific organizational goals and technological contexts.

## 1. Introduction

Health Information Systems (HISs) have long been recognized as essential tools in healthcare delivery and public health [1]. An HIS refers to any system or framework that facilitates the communication, processing, or transmission of health-related information via electronic means to improve health outcomes [2]. The adoption of HIS has grown rapidly across healthcare settings, driven primarily by two key factors: (1) the escalating burden of chronic diseases, with associated costs rising significantly, and (2) the increasing demand for improved quality and safety in healthcare delivery [3,4,5,6]. These drivers have led to substantial investments in HISs to support timely information sharing and enhance clinical decision-making.

Healthcare’s ongoing transformation is largely driven by demographic shifts—including aging populations and rising chronic illnesses—alongside cultural changes, scientific and technological advancements, and a heightened emphasis on quality and safety [6,7,8,9,10]. The core function of HIS is to manage data related to healthcare planning, monitoring, coordination, and decision-making [11]. Real-time access to clinical data has improved documentation, reduced redundant services, and enhanced decisions about patient care [12,13]. HIS systems are designed to support clinicians in accessing and utilizing patient information efficiently while facilitating the sharing of quality healthcare data [14,15,16]. Effective HIS implementations have consistently been proven to improve patient care and assist healthcare professionals and managers in making informed decisions [17,18].

In pursuit of better management practices, many healthcare organizations have turned to Maturity Models, despite some ongoing criticism of these frameworks [19,20]. The fundamental principle of Maturity Models is that organizations (or their components) evolve through progressive stages toward higher levels of capability and performance [21,22]. Maturity plays a key role in the value organizations can derive from practices like project management [23,24], and improvements often require a structured action plan [25,26].

Governments worldwide have invested heavily in enhancing process management and reforming health systems to promote transparency, improve quality and safety, increase patient satisfaction, and, critically, control costs [27]. However, there is still no clear consensus on which capabilities hospitals must acquire to become truly process-oriented organizations, nor is there agreement on the specific sequence of steps to achieve such developments [28,29]. Inspired by successful models in other industries, the healthcare sector has begun adopting organizational frameworks such as patient-centered models [30,31]. Empirical studies have shown that process orientation significantly enhances hospital performance [32].

This article aims to explore and synthesize the various Maturity Models applied to Health Information Systems, highlighting their relevance and application across different healthcare contexts.

## 2. HIS Implementation Challenges

Research has consistently shown that Health Information System (HIS) projects often face high failure rates, particularly within hospital settings [33,34]. Despite the substantial and continually increasing financial investments in healthcare HIS worldwide, these projects remain risky. Although technological innovations in healthcare promise significant benefits, they also present considerable challenges. Consequently, it is essential for organizations to adopt structured project-evaluation processes and benefit-management frameworks to maximize the realization of anticipated outcomes from these investments [35,36].

Numerous studies highlight the prevalence of HIS project failures. For instance, Bagherian and Sattari [37] report a significant proportion of such projects failing to meet their objectives. Heeks [38] estimates that 35% of HIS projects are complete failures, 50% achieve only partial success, and just 15% are fully successful. Kaplan and Harris-Salamone [33] also observed failure rates exceeding 30% in large-scale HIS initiatives.

The implementation of Health Information System (HIS) projects in healthcare has often resulted in wasted financial resources, particularly due to the acquisition of large-scale systems that ultimately prove ineffective [30,39]. Unlike projects in other industries, HIS implementations present unique challenges. These stem from the complexity of the healthcare environment, the diversity of systems and devices that must function cohesively, and the critical need for integration and interoperability. Moreover, meeting the varied expectations of stakeholders regarding what constitutes project success adds another layer of difficulty [40]. *Why do HIS implementations fail in health organizations?*

Healthcare projects are inherently complex, and their success largely depends on the quality of available information. This includes the accuracy, completeness, and consistency of data—particularly within electronic health records. Aerts [41] offers a comparative analysis of telemedicine Maturity Models, illustrating how capability gaps often contribute to delayed adoption in rural areas. Poor-quality data, whether inaccurate or incomplete, can result in diagnostic errors, ineffective treatments, and compromised patient care, underscoring the essential role of data quality in healthcare [42]. To deliver successful projects, organizations must possess three essential categories of skills [43,44]:

1. Technical skills—encompassing a broad spectrum of expertise, including technical knowledge, practical experience, and functional capabilities;
2. Project management skills—involving the knowledge, methodologies, and competencies required to effectively manage Health Information System (HIS) projects;
3. People and organizational skills—referring to the interpersonal and organizational abilities needed to engage and collaborate effectively with the diverse stakeholders involved in HIS initiatives.

Project failures in healthcare are often attributed to a range of interrelated factors [45], including the following:

1. Lack of senior management commitment, which is frequently incomplete or entirely absent [46];
2. Challenges in engaging healthcare professionals and insufficient attention to end-user needs [47];
3. Inaccurate specification of requirements, leading to system misalignment with clinical needs [48];
4. An absent or poorly managed change process, which undermines adoption efforts [49];
5. Limited understanding of the complexity of healthcare systems, resulting in ineffective implementation strategies [46];
6. Insufficient investment in human resources, which hampers the capacity to support and sustain digital initiatives [50];
7. Inadequate training, a critical factor influencing health professionals’ willingness and ability to adopt and integrate information systems into clinical practice [51].

A positive organizational culture can significantly influence the development of superior project management practices. A culture that promotes collaboration, trust, and open communication, for example, can lead to more effective teamwork, better risk management, and ultimately, more successful projects [52]. These profound changes implicate important ethical challenges. However, a complementary approach involves identifying and leveraging Critical Success Factors (CSFs) that enable effective implementation and sustainable use. According to Pereira et al. [53], CSFs—such as strong leadership, user involvement, change management readiness, and technological infrastructure—can serve as guiding pillars for aligning HIS deployment with strategic objectives. Within this context, Maturity Models offer a structured framework to assess how well these CSFs are embedded in an organization’s practices, providing a roadmap for capability enhancement and continuous improvement.

## 3. Maturity Models for HISs

Organizations are increasingly adopting Maturity Models to enhance management practices despite facing criticism [54,55]. These models, which assess an organization’s capabilities against a predefined set of stages, provide a framework for improvement and alignment across different areas [56]. While lauded for their ability to guide improvement and foster a common language, Maturity Models have also been criticized for being static, potentially overlooking context-specific needs, and lacking empirical evidence of their direct link to performance [57]. 

Maturity Models offer a structured approach to assessing and improving an organization’s management practices [58]. They outline progression through different stages, from initial, ad-hoc processes to mature, optimized ones. Friedman and Flynn [59] proposed a maturity grid assessment tool tailored for learning networks, exemplifying how structured models can guide development across distributed healthcare environments. This framework helps organizations identify areas for improvement and develop a roadmap for achieving higher levels of maturity [19].

Despite criticism, Maturity Models are widely adopted because they offer several benefits. They can improve project management capabilities, increase customer satisfaction, lead to higher returns on investment, and enhance schedule and budget sustainability [60]. Organizations need to consider their specific context and adapt the Maturity Model to their unique needs and challenges. A one-size-fits-all approach may not be effective [61]. While Maturity Models offer a valuable framework for improvement, organizations should use them with caution, considering their limitations and adapting them to their specific contexts [55].

Several Maturity Models have been developed, particularly in the health sector, although many of them are still at an early stage of development [19]. The concept of maturity considers that more mature organizations will have more systematic and constant performances, while less immature organizations achieve their results largely through the redoubled effort of individuals through approaches that they create and use spontaneously [62]. Hospitals with a more mature information systems (IS) infrastructure tend to have more formalized IS planning and control processes [63]. This is because a more mature IS infrastructure often signifies a greater reliance on and integration of technology across various hospital functions [64]. This reliance necessitates more structured approaches to planning, implementing, and managing these systems to ensure efficiency, reliability, and the alignment with overall organizational goals [65].

Process maturity refers to the degree to which a specific process is clearly defined, managed, measured, controlled, and effective [66]. To clarify these different uses of the term “maturity”, it is helpful to distinguish between three core dimensions. Technical maturity refers to the readiness and integration of IT infrastructure, including hardware, software, and digital tools [64,67]. Organizational maturity encompasses leadership commitment, governance structures, the institutional culture, and staff capability to adapt to information systems [54,58]. Process maturity reflects the extent to which clinical and administrative workflows are formalized, standardized, and continuously optimized [66,68]. Recognizing these dimensions supports a more targeted application of Maturity Models within healthcare settings.

In an effort to reduce costs and improve productivity, governments have increasingly implemented enterprise resource planning (ERP) systems and process management systems [68]. To identify and leverage the strengths and weaknesses of organizational projects, a wide range of Maturity Models (MMs) have been developed in recent years [69].

MMs have become a significant topic in management research. They are typically defined as multi-stage conceptual frameworks that describe common developmental patterns in organizational capabilities. These models are grounded in the principle of natural evolution, portraying organizational development as a progression from lower to higher levels of maturity through a structured sequence of phases [70].

While research has increasingly focused on refining complex and rigorous approaches to MM development, there is a growing emphasis on effectively applying these models in complex organizational environments, such as hospitals [71]. Numerous models have been introduced to assess and describe the current levels of information system (IS) adoption in the healthcare sector [72].

The capability of an organization’s IS infrastructure is a key factor in defining the sophistication of its IS services, particularly in supporting a wide range of applications within hospitals [73,74,75]. The completeness of this infrastructure is generally proportional to the maturity level of a hospital’s Health Information System (HIS) [67,76]. Many HIS studies have highlighted system integration as a major challenge in the development of IS in healthcare organizations [37,73]. To address this issue, several standards have been established to facilitate data exchange between healthcare providers [77].

Overall, there is a growing consensus that organizations will increasingly adopt Maturity Models as strategic tools to stimulate and guide the advancement of their IS capabilities [78].

## 4. Methodology

The article adopts a methodological structure grounded in a comprehensive literature review to establish a robust documentary foundation [79]. Specifically, it follows a scoping review methodology, guided by the framework proposed by Tranfield et al. [80] and aligned with the PRISMA Extension for Scoping Reviews (PRISMA-ScR) [81]. The review process was conducted in five key steps:

- Definition of search terms, keywords, and their combinations to guide the review criteria.
- Identification of relevant publications containing the specified keywords.
- Evaluation of the identified literature and selection of works that met the inclusion criteria.
- Extraction of relevant information from the selected studies.
- Synthesis and analysis of the extracted data.

The initial search was conducted across major academic databases, including the AIS Electronic Library, ISI Web of Knowledge, SCOPUS, Springer, Elsevier/ScienceDirect, and the IEEE Computer Society Digital Library. To ensure broader coverage and capture potentially overlooked studies, additional searches were also performed using Google Scholar and general web-based queries.

### 4.1. Inclusion and Exclusion Criteria

To ensure consistency, transparency, and replicability in the selection of sources, predefined inclusion and exclusion criteria were applied during the screening and full-text review stages.

Inclusion criteria:

- Peer-reviewed journal articles or conference papers.
- Published between 2000 and 2025.
- Written in English.
- Explicit focus on Maturity Models within the context of healthcare, digital health, or Health Information Systems (HISs).
- Articles addressing the development, application, or evaluation of Maturity Models.
- Studies presenting practical, empirical, or theoretical contributions related to HIS maturity.

Exclusion criteria:

- Articles not related to Maturity Models or not applied in healthcare settings.
- Conceptual work lacks empirical grounding or practical application.
- Studies focused on general IT or other sectors (e.g., manufacturing, education) without healthcare-specific adaptation.
- Non-peer-reviewed publications, editorials, theses, or opinion papers.
- Non-English language sources.
- Duplicate records.

### 4.2. Search Strategy and Criteria

As a quality criterion, all studies that mentioned Maturity Models—either directly or indirectly—and clearly identified contextual factors such as motivations, objectives, outcomes, and benefits were included (see Table 1).

Each Maturity Model identified was characterized based on its description, scope, developmental stages and their characteristics, sizes, influencing factors, and the methods employed in both its development and validation processes. After the full analysis, a total of 50 models were selected, which are described in the following sections.

## 5. Classification of Maturity Models

To better understand the breadth and focus of existing Maturity Models applied to Healthcare Information Systems (HIS), this section presents a classification based on their core functional orientation. Through an in-depth literature review, we identified 50 Maturity Models, which—despite their unique characteristics—tend to cluster around four main thematic categories:

Process-Oriented Models: These models concentrate on evaluating and improving healthcare organizations’ internal workflows, project governance, and management practices. Examples include the Capability Maturity Model Integration (CMMI) and the Business Process Orientation Maturity Model (BPOMM).

- Technology-Focused Models: These assess the maturity of technological infrastructure, IT services, and system adoption levels. They provide guidance on infrastructure development, IT governance, and digital capability. Notable examples include the Healthcare IT Maturity Model (HIT-MM) and the Infrastructure Adoption Model (INFRAM).
- Specialized Domain Models: These target specific healthcare functions or domains, such as telemedicine, usability, interoperability, or public health coordination. They are typically designed for tailored applications, such as the Telemedicine Service Maturity Model (TMSMM) or the Interoperability Maturity Model (IMM).
- Data and Analytics Models: These models address the organization’s capacity to manage, analyze, and utilize healthcare data effectively. They focus on data quality, analytics maturity, and decision support capabilities. Prominent examples include the Healthcare Analytics Adoption Model (HAAM) and the Business Intelligence Maturity Model (BIMM).
- Policy-Oriented Models: These models address the development, implementation, and institutional maturity of health-related public policies, especially in intersectoral and government settings. They are primarily intended for use by policymakers, administrators, and public institutions aiming to improve population health through systemic governance, for example, the Maturity Model for Health in All Policies (MMHiAP).

Each of these models conceptualizes maturity as a progressive continuum—ranging from rudimentary, ad hoc implementations to advanced, optimized, and integrated systems. The classification framework serves to highlight the differences in the emphasis, design, and intended use of the models. Accordingly, the models are also compared across key analytical dimensions such as scope, domain specificity, practical applicability, and ease of implementation, to assist stakeholders in selecting the most suitable model for their organizational needs.

### 5.1. Process-Oriented Models

**(1) Capability Maturity Model Integration for Services (CMMI)**

One of the most widely recognized Maturity Models (MMs) is the Capability Maturity Model Integration (CMMI), originally developed within the field of software engineering to assess the maturity of software development practices [82]. Over time, its application has expanded to various other domains. CMMI serves as a process improvement framework and assessment tool, structured around five maturity levels. Each level is defined by specific cumulative requirements, and organizations are evaluated using standardized question sets and assessment criteria to measure the maturity of their product development processes.

CMMI incorporates a self-assessment component that highlights an organization’s best practices in key process areas and guides them on how to enhance their capabilities as they progress toward higher maturity levels [82]. Specific CMMI variants cater to different areas: CMMI for Development (CMMI-DEV) aligns with medical device standards by emphasizing process performance and continuous improvement, addressing areas such as Design Controls and Production and Process Controls. CMMI for Acquisition (CMMI-ACQ) focuses on procurement and supply chain processes, supporting quality system elements like purchasing controls relevant to medical devices. Meanwhile, CMMI for Services (CMMI-SVC) provides guidance on service delivery and management, applicable to both internal and external health service operations. As such, the objectives and practices of CMMI-SVC are particularly relevant to organizations involved in delivering healthcare services [82].

**(2) Business Process Orientation Maturity Model (BPOMM)**

The Business Process Orientation Maturity Model (BPO-MM) was developed to assess the maturity of employee-related processes within large hospital units [83]. This model enables hospitals to evaluate their progress toward achieving a process-oriented approach. It outlines a series of developmental stages—ad-hoc, defined, linked, and integrated—that organizations must navigate to attain full process maturity [84,85]. The model also facilitates benchmarking, allowing healthcare facilities to compare their process maturity against competitors or similar organizations.

The BPO-MM assessment tool consists of 35 questions covering seven key dimensions, which are grouped into two categories: BPO Components and BPO Impacts [83]. The BPO Components assess the degree of process orientation within the organization and include three dimensions: Process Vision (four items), Process Work (three items), and Process Management and Measurement (four items). The BPO Impacts encompass the remaining four dimensions, focusing on the extent to which process orientation contributes to improved organizational performance and sustainable health outcomes.

The literature highlights several benefits associated with higher process maturity, including cost savings from more efficient task execution, shorter cycle times, enhanced customer focus, better organizational integration, increased flexibility, improved customer satisfaction, and the elimination of redundant products and duplicated activities [86].

**(3) Process Management Maturity Model (PMMM)**

Assessing an organization’s level of IT maturity requires more than simply evaluating its technological infrastructure. Factors such as employee attitudes toward technology adoption also play a crucial role and should be included in maturity assessments. Nolan’s staging model is one of the well-known frameworks that captures this broader perspective. In the healthcare sector, however, there remains a lack of consensus on the specific capabilities and developmental stages that hospitals must achieve to become truly process-oriented organizations [87].

Effective process management in hospitals demands a strong focus on cultural and structural capabilities, extending beyond the conventional reliance on IT systems and automation prevalent in other industries. This distinction arises because healthcare is fundamentally centered on human interaction, social dynamics, and the individualized needs of patients, making its processes inherently more complex and people-driven [28].

The conceptual foundation of the Project Management Maturity Model (PMMM) is structured around five key capability dimensions:

1. Culture—Encompasses organizational practices related to communication, leadership, and the overall receptiveness to change and collaboration.
2. Strategy—Refers to the guiding principles and strategic alignment necessary for the effective implementation and evolution of process management within healthcare institutions.
3. Structure—Includes the organizational architecture, governance mechanisms, and role definitions that support process-based approaches.
4. Practices—Represents the operational methodologies and standardized procedures that are central to consistent and effective process management.
5. Information Technology (IT)—Captures the extent to which hospital IT systems facilitate the seamless integration and continuity of end-to-end patient care processes.

This multi-dimensional approach is also reflected in more recent models, such as the OPM3-based maturity study conducted by Gomes et al. [88], which emphasizes project portfolio alignment, capability development, and strategic governance within healthcare organizations. Moreover, the Maturity Model (MM) can be further refined by incorporating individual scores for each component of the Business Process Orientation Maturity Model (BPOMM), along with their respective impacts. The literature highlights several benefits associated with higher levels of process maturity. These include cost savings through more efficient work execution, reduced cycle times, enhanced customer focus, improved cross-functional integration, increased organizational flexibility, and higher levels of customer satisfaction. Additionally, process maturity contributes to the elimination of redundant and duplicated activities, fostering overall operational efficiency [83].

**(4) Information Management Maturity Assessment Program (IMMAP)**

The Information Management Maturity Assessment Program (IMMAP) was introduced by the Public Record Office Victoria (PROV), Australia, in January 2016. Conducted biennially, the program requires government departments and key agencies to assess their information management (IM) practices using PROV’s Information Management Maturity Measurement (IM3) tool. The primary objectives of the IMMAP are to accomplish the following [89]:

Provide a framework for identifying and initiating information management improvement opportunities within and across government entities.

Establish an evidence base to guide the strategic direction and priorities for IM decision-makers across the Victorian Government.

The IM3 tool evaluates an organization’s current information management maturity through a structured questionnaire and supporting documentation. It is designed to achieve the following:

- Measure performance against the Victorian Government’s IM standards.
- Assess an organization’s alignment with information management best practices.

While the tool is primarily intended for use by information managers, the assessment process encourages collaboration. Multiple stakeholders—such as experts in information security, data management, and records management—should contribute to ensure a comprehensive evaluation. This collaboration may involve meetings, interviews, surveys, or workshops.

By completing the IM3 assessment, organizations can pinpoint areas for improvement, identify trends in information management maturity over time, and gather actionable data (e.g., tables and charts) to inform their information strategies. After each assessment cycle, PROV publishes an Information Management Maturity Current State Assessment Report, which includes the following:

- Maturity level ratings for each IMMAP participant.
- Average maturity levels across key IM areas.
- Recommendations for future IM improvement initiatives.

**(5) Networkability Maturity Model (NMM)**

The Networkability Maturity Model is a general-purpose maturity framework that aligns with standard maturity levels and is designed to assess the key factors influencing an organization’s ability to engage in effective networking—termed *networkability*. In the context of healthcare providers’ administrative processes, the model includes a structured framework consisting of six components. Two of these components—strategy and system—span horizontally across the model’s foundational layers, while the remaining four components focus on strategy execution and extend vertically across the Business Engineering layers. This layered approach facilitates a comprehensive assessment of how well administrative processes support collaboration, integration, and interoperability within networked healthcare environments (Table 2) [90].

**(6) General Practice Information Maturity Model (GPIMM)**

The General Practice Information Maturity Model (GPIMM) defines maturity levels for information management within primary care settings. At its core, the model offers a snapshot of how developed an organization’s information processes are—particularly in the context of the transition from paper-based records to electronic health records (EHRs) within the UK’s National Health Service (NHS). Primary care in the NHS has traditionally been organized around small practices, often comprising clusters of one to six physicians supported by a team of healthcare professionals and administrative staff. These practices were overseen by Primary Care Trusts (PCTs), leading to a highly diverse and frequently fragmented system. The GPIMM was designed to help standardize and assess the maturity of such varied organizational structures in their adoption and use of digital Health Information Systems. There was a wide range of organizational maturity and personal capacity to move towards the declared national goal of paperless by 2005 [91].

**Figure 1:**
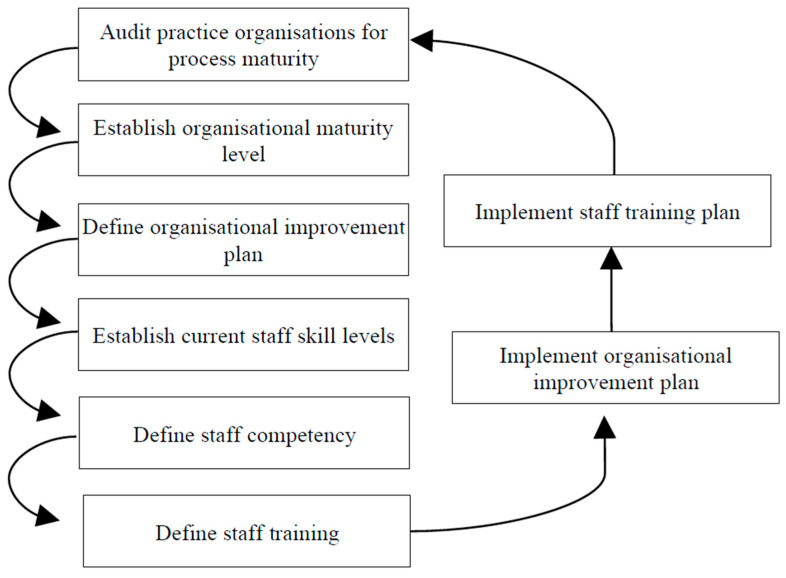
The organizational change process.

The General Practice Information Maturity Model (GPIMM) was created through a structured methodology (Figure 1), with its processes automated using dedicated tools that implement the model. These tools support organizational audits and assess current skill levels, tasks typically carried out by a practice facilitator from the managing organization, such as Primary Care Trusts (PCTs). During on-site visits, the practice facilitator conducts a process audit using a standardized questionnaire. After the assessment, the tool calculates the practice’s maturity level and provides targeted recommendations for key improvement areas. Additionally, the tools can produce reports for fund managers, presented in both textual and graphical formats [91].

### 5.2. Technology-Focused Models

**(7) HIMSS Analytics Electronic Medical Record Adoption Model (EMRAM)**

The Electronic Medical Record Adoption Model (EMRAM) is a globally recognized standard developed by HIMSS Analytics in 2005. It measures the adoption and utilization of electronic medical records (EMRs) in healthcare institutions, categorizing hospitals along an eight-stage maturity scale (0–7). EMRAM employs standardized methodologies and algorithms to automatically rank hospitals worldwide based on their EMR capabilities and is used by over 9000 hospitals globally [92]. The eight-stage (0–7) model evaluates the adoption and utilization of Electronic Medical Record (EMR) functions, guiding healthcare organizations toward a near paperless environment where technology is leveraged to enhance patient care [93]. An EMR is a complex, electronic patient record system typically implemented through a series of hierarchical, progressively advanced stages [94].

By advancing through the stages of the Electronic Medical Record Adoption Model (EMRAM), healthcare providers transition patient records to electronic formats accessible across both inpatient and outpatient settings. This enables clinicians to document, monitor, and manage patient care more efficiently.

The results from an EMRAM assessment help organizations identify critical improvement areas, inform the development of their IT strategy, and align digital initiatives with broader organizational goals [95].

**(8) Electronic Healthcare Maturity Model (eHMM)**

The Electronic Healthcare Maturity Model (eHMM) incorporates all service providers involved in healthcare processes and is designed to be adaptable to specific providers at any maturity stage. It also accommodates varying maturity levels across different business processes, offering flexibility for diverse healthcare environments [96]. The model defines seven distinct maturity levels to guide healthcare organizations through a roadmap of continuous process improvement. These stages represent the evolution from initial, ad hoc operations to optimized and fully integrated electronic healthcare systems.

Each maturity level is characterized by unique capabilities and strategic focus areas, providing organizations with a structured framework for assessment and development. eHMM contributes to the Maturity Model discussion by emphasizing the importance of aligning technological progress with organizational readiness and capacity for change. This alignment ensures that digital health systems not only mature technically but also evolve in terms of governance, interoperability, and data utilization to support integrated care delivery. eHMM offers a holistic approach to digital transformation in healthcare, promoting strategic planning, improved clinical outcomes, and organizational agility [96,97].

**(9) IDC MaturityScapes (IDC-MS)**

IDC MaturityScapes (IDC-MS) were developed by IDC Health Insights to provide a structured framework for evaluating organizational maturity. The model outlines five stages—from the ad hoc and unstructured to the optimized and systematized—helping managers assess their current capabilities, identify gaps, and plan targeted improvements to maintain a competitive advantage [98]. The framework is designed to adapt to shifts in the information systems ecosystem, such as technological advances, the integration of platforms like mobile and social applications, or the evolution of existing tools. It supports decision-makers in aligning business objectives with IT strategies and identifying priority areas for investment in people, processes, and technologies [99]. IDC-MS is often used to inform IT governance dashboards, align strategic initiatives, and measure progress against industry benchmarks. Its five dimensions span technological, organizational, and human factors and are structured to support holistic data maturity and long-term digital transformation strategies [98].

**(10) IDC Mobility Maturity Model (IDC-Mobility)**

The healthcare industry is experiencing a mobile-driven transformation, fueled by the consumerization of technology and the digitization of patient health information [100]. Patients increasingly expect to use mobile devices to engage with their healthcare providers and insurance plans, as well as to actively manage their own health [100].

The Maturity Model for Mobile in Healthcare outlines five stages of mobile maturity: ad hoc, opportunistic, repeatable, managed, and optimized. The IDC-Mobility for Healthcare framework provides essential components for creating an enterprise mobility roadmap. This framework is designed to help healthcare organizations perform the following:

- Assess their current mobility capabilities and maturity;
- Establish a baseline to define short- and long-term goals and improvement plans;
- Prioritize investments in mobility-related technologies, staff, and infrastructure;
- Identify maturity gaps across business units or between business operations and IT functions [101,102].

**(11) IDC Healthcare IT Maturity Model (IDC-HIT)**

IDC (Health Industry Insights) has created a Maturity Model outlining five stages of hospital information system (SI) development: basic HIS, advanced SIS, clinical SIS, digital hospital, and virtual hospital [103]. Each stage builds upon the capabilities of the previous one, representing a progressive evolution of the hospital IT infrastructure. The Healthcare IT (HIT) Maturity Model has been widely applied by IDC to evaluate the maturity levels of Hospital Information Systems (HISs) across the globe [104].

**(12) Healthcare Information Technology Maturity Model (HITMM)**

HIMSS Analytics USA/Europe and the Innovation Value Institute have collaborated to develop a leading program that helps hospitals strengthen their organizational IT capabilities to achieve improved eHealth outcomes [105]. The Healthcare IT Maturity Model (HIT-MM) is specifically designed for CIOs and senior IT leaders who are responsible for managing clinical eHealth systems, as well as traditional IT infrastructure. The program’s assessments provide a clear basis for senior management to make informed decisions about enhancing critical IT capabilities—capabilities viewed as essential for supporting and improving Hospital Information Systems (SISs) and related services [105].

HIT-MM enables hospitals to assess both the maturity of their healthcare IT services and the organizational resources required to deliver and sustain these services. This integrated approach reveals dependencies that may hinder the strategic planning, deployment, and execution of clinical IT services. It also links the adoption of EMR systems with the maturity of underlying organizational IT resources [105]. Recent studies highlight the importance of such unified maturity frameworks in connecting technological capability with improved healthcare outcomes, particularly within the rapidly evolving digital health landscape [54].

**(13) NHS Infrastructure Maturity Model (NIMM)**

The National Infrastructure Maturity Model (NIMM) framework assists NHS IT organizations in conducting objective assessments of their IT infrastructure and identifying key areas for maturity improvement. The framework is structured into 13 categories, 74 capabilities, five perspectives, and multiple key performance indicators (KPIs), with each element assessed across five maturity levels [106].

Organizations are not required to address all features simultaneously. Instead, they are encouraged to review the available features, set priorities based on current needs, and focus their efforts on a selected subset. Each category comprises multiple features that guide the assessment toward a specific domain of IT infrastructure.

Capabilities within the framework are grouped under broader perspectives. Each perspective is linked to several KPIs, which are used to evaluate the maturity of the related capabilities. By organizing metrics into perspectives, the NIMM framework enables IT teams to gain a holistic understanding of their infrastructure, extending beyond purely technical considerations to include strategic and operational viewpoints.

**(14) PACS Maturity Model (PMM)**

PACSs have become a core part of modern healthcare delivery, serving as a critical infrastructure for digital imaging diagnostics and information management systems [107]. The PACS Maturity Model (PMM) evaluates the process maturity of hospitals in relation to their use of PACS. Developed as both a descriptive and normative framework, the PMM guides evaluations and strategic planning across five levels of maturity [108].

This model helps hospitals understand their strategic development goals and maturity targets concerning PACS, electronic patient records, and other health information systems [108]. The PMM integrates three key concepts [109]:

1. PACS maturity, defining PACS and its components;
2. Alignment of PACS, focusing on how PACS integrates with the hospital’s organizational structures;
3. PACS performance, reflecting the added value PACS brings to healthcare delivery.

In addition to strategic planning, the PMM serves as a practical tool for organizational assessments, ongoing performance monitoring, and benchmarking against industry standards.

**(15) Hospital Information System Maturity Model (HISMM)**

The Health Information System Maturity Model (HISMM) is designed to address the inherent complexity of Health Information Systems (HISs) and serve as a practical tool to support the challenging responsibilities of HIS management. Structurally, HISMM follows the conventional Maturity Model framework, organized as a matrix of progressive maturity stages and six key influencing factors: data analysis, strategy, people, electronic medical records, information security, and systems/IT infrastructure—identified as critical elements in healthcare environments [110].

These factors act as descriptors that define each stage of maturity and outline the criteria required to progress to the next level. HISMM is structured as an evolutionary scale, with clearly defined, measurable transitions between stages. Each maturity level is characterized by a specific set of attributes, and a Health Information System (HIS) is considered to have reached a given level once it demonstrates those attributes. The model describes six stages of maturity: Adhocracy, Starting Foundations, Centralized Dictatorship, Democratic Cooperation, Entrepreneurial Opportunity, and Integrated Relations [94].

Hospitals can use the HISMM framework to perform the following:

- Identify their current maturity stage;
- Determine the next achievable stage of maturity;
- Recognize the attributes required to advance to the next level.

Importantly, HISMM supports more informed decision-making regarding the allocation of limited resources, especially to improve the organization’s information security environment. The model is particularly valuable for smaller or less mature healthcare institutions, as it requires minimal effort to conduct a comprehensive assessment [111]. By applying HISMM, healthcare organizations can implement effective information security programs that enhance data integrity and the reliability of their systems, strengthen regulatory compliance, and reduce the risks associated with both internal and external threats. The healthcare sector stands to benefit greatly from improved information security maturity—not only through significant cost savings, potentially amounting to hundreds of millions of dollars, but also by safeguarding patient safety and saving lives [111].

Furthermore, the model promotes better institutional decision-making by helping organizations understand the maturity of their information security environments. HISMM is a cost-effective and easy-to-administer solution that minimizes the need for external consultants or large internal teams. It also facilitates the sharing of best practices across various healthcare organizations, regardless of their size or type [111].

**(16) Forrester “Meaningful Use” Model**

A wide range of systems, organizations, and processes are used to manage medical records and the associated workflows, with healthcare providers taking diverse approaches and progressing at varying speeds to address these challenges. This variability has given rise to a three-phase Maturity Model that uses content management, collaboration, and workflow technologies as foundational elements for advancing medical record management.

Forrester’s Maturity Model provides a framework that enables healthcare vendors to assess their current capabilities in content, collaboration, and workflow and, more importantly, to chart a roadmap for progressing to the next maturity phase [112]. The three phases of the model are as follows:

- Phase 1: Patient records are primarily paper-based or image-based. Most healthcare providers operate at this stage, where patient information management is viewed largely as a content- or records-management challenge.
- Phase 2: Access to standalone electronic repositories improves. Providers at this stage store more patient data within Electronic Medical Record (EMR) systems and reduce their reliance on paper records.
- Phase 3: Role-based access to a fully digital medical record is achieved. Providers exchange electronic data with other healthcare organizations, patients, and administrative systems. At this level, content is structured to support results-driven analysis.

According to Le Clair [112], the transition through these phases is influenced by four key dimensions: access, interoperability, content resources, and planning and strategy. These factors shape how effectively a healthcare organization can advance toward comprehensive, digital, and collaborative medical record management.

**(17) IGHealthRate™ (IGHR)**

Iron Mountain’s Enterprise Information Governance (EIG) framework provides a comprehensive and adaptable approach to information governance (IG), enabling organizations to manage information efficiently and cost-effectively [113]. Its objectives include the following:

- Governing all types of information—regardless of format or location—in a consistent and coordinated way;
- Securing information throughout its lifecycle and across the organization’s ecosystem, covering both data and IT governance;
- Addressing data privacy and integrity requirements, ensuring compliance while maximizing the value derived from information.
- This enterprise-wide framework enables organizations to treat information as a strategic asset, supporting a wide range of needs, including strategic planning, regulatory compliance, legal obligations, risk management, and environmental responsibility.

The EIG framework is operationalized through IG-HealthRate™, a healthcare-focused assessment tool. IG-HealthRate™ evaluates IG maturity using 10 organizational competencies and more than 85 indicators, providing a detailed understanding of an organization’s information governance capabilities across its ecosystem. Recognized as the healthcare industry standard for IG capability assessment, IG-HealthRate™ examines how information is created, used, classified, secured, and disposed of—whether in physical or digital form.

Measuring IG maturity enables organizations to establish a clear baseline for improvement, aligned with key digital health objectives. The ten competencies assessed by IG-HealthRate™ are as follows:

- IG Structure
- Strategic Alignment
- Enterprise Information Management
- Privacy and Security
- Legal and Regulatory Compliance
- Data Governance
- IT Governance
- Analytics
- IG Performance
- Awareness and Adherence

These competencies provide insight into the organizational structures and processes needed for the adoption, implementation, and ongoing development of effective information governance practices. The EIG framework is designed to be scalable and flexible, making it applicable to organizations of all sizes and types. Its adaptable nature supports a progressive path toward continuous improvement and greater IG maturity.

**(18) Public Health Information Technology (PHIT)**

PHIT has the potential to improve the effective and efficient use of information in achieving public health objectives. Information technology Maturity Models have been extensively used in other domains to guide IT assessments and planning, but a model tailored specifically to public health departments had been lacking [114]. The development of the PHIT Maturity Index was supported by an extensive literature review of prior work on information systems maturity and Maturity Models within the context of public health systems and services. The PHIT Maturity Index comprises four main categories: (1) Scale and Scope of Use of PHIT, (2) PHIT Quality, (3) PHIT Human Capital, Policy, and Resources, and (4) PHIT Community Infrastructure—along with fourteen sub-dimensions. This model represents a practical framework to assist public health system stakeholders, particularly health departments, in evaluating their information technology deployment decisions. As benchmark data become available, the index may also enable comparative assessments and the potential correlation between IT maturity, multi-agency interoperability, and population health outcomes [114]. The PHIT Maturity Index has been further validated and adapted in different country contexts. For example, Wijayarathne et al. [115] modified and empirically tested the tool for public health information system implementations in Sri Lanka, demonstrating its flexibility and relevance across diverse health systems.

**(19) IT Maturity Model for Smart City Services in Emerging Economies (FSCE2)**

The FSCE2 framework provides a structured approach for developing an IT Maturity Model aimed at guiding the planning, design, and implementation of smart city services. Its primary objectives are to qualitatively define and quantitatively measure the maturity levels of critical IT dimensions within smart cities—specifically IT governance, IT services, data management, and infrastructure—and to offer a practical, adaptable model that meets the unique needs of any territory seeking to develop or enhance smart city services [116].

The framework consists of three core components:

- A conceptual model of smart city services;
- Clearly defined IT dimensions and indicators;
- Distinct IT maturity levels.

These components are designed for practical, context-sensitive applications, enabling cities to implement smart services in a way that directly impacts citizens’ quality of life. A city qualifies as a smart city under FSCE2 if it has at least one IT-driven initiative that enhances citizen services and integrates across four core areas: the opportunity economy, infrastructure, governance, and a sustainable environment [116].

The FSCE2 framework evaluates IT maturity across four key dimensions, with relevant healthcare examples:

IT Governance → eHealth Strategy and Policy Alignment:

Strong IT governance is critical for managing EMR systems, telemedicine platforms, and regulatory compliance (e.g., HIPAA, GDPR).

Use case: Local governments aligning smart health initiatives—such as vaccination programs or chronic disease management—with citywide IT governance frameworks.

IT Services → Telehealth, mHealth, and Remote Monitoring:

This dimension focuses on the service delivery platforms for telemedicine, e-prescriptions, and AI-assisted triage.

Use case: Cities deploying mobile health units or virtual consultations using service models aligned with FSCE2.

Data Management → Electronic Health Records (EHR) and Interoperability:

Emphasizing structured data collection, integration, and analytics essential for population health management and clinical decision support.

Use case: Public health agencies leveraging integrated EHRs for real-time outbreak monitoring.

Infrastructure → Smart Hospitals and IoT Devices:

Covers the technological foundation supporting wearable health devices, remote ICUs, smart ambulances, and other IoT-enabled services.

Use case: Smart hospital wards interconnected with citywide utilities like power, water, and emergency services to form a resilient ecosystem.

The framework defines three progressive stages of IT maturity:

- Level 1: Integrated—Information is collected, stored, and integrated, making it readily available for smart services.
- Level 2: Analytically Managed—Advanced analytics (descriptive, predictive, prescriptive) are applied, enabling the generation of smart dashboards for decision-making.
- Level 3: Optimized Automated—Beyond integration and analytics, this level incorporates artificial intelligence to automate processes, enhancing decision-making, operational efficiency, and system performance.

FSCE2 provides a flexible, scalable roadmap for cities seeking to optimize their digital infrastructure, including healthcare services, through smart technologies and data-driven governance.

**(20) Cloud Security Capability Maturity Model (CSCMM)**

The Cloud Security Capability Maturity Model (CSCMM) is structured around two key dimensions: security domains and maturity levels. The framework includes twelve security domains, each comprising a set of cybersecurity practices that serve as achievement objectives tailored to that domain [117]. The model defines four maturity levels, enabling organizations to assess and improve their cybersecurity posture progressively.

CSCMM is adaptable to various cloud service models (e.g., Infrastructure Platform Software as a Service—IPSaaS) and deployment types, including public, private, and hybrid clouds. It offers practical guidance for implementing and enhancing an organization’s cloud cybersecurity capabilities [118].

For senior management, CSCMM provides a structured assessment of their cloud security posture, supporting informed decision-making regarding the business strategy and direction. For security professionals and practitioners, the model’s quantitative metrics enable the implementation of proactive security measures and facilitate timely, responsive actions to emerging threats [118].

**(21) Healthcare Cloud Security Maturity Assessment Framework (HCSMAF)**

The Healthcare Cloud Security Maturity Assessment Framework (HCSMAF) was developed to help healthcare organizations assess their security posture through a user-friendly and healthcare-focused approach, emphasizing ease of use and cloud-centric security controls [119]. To deliver meaningful impact, the tool was designed and implemented as a cloud-based application, providing a practical and accessible solution for healthcare environments.

HCSMAF operates on a centralized computing model, using a client–server architecture where data is transmitted over an HTTP channel. The application is hosted in a cloud environment on the Google Cloud Platform (GCP), running on a Virtual Machine (VM) [120]. GCP was selected due to its combination of cost-effective long-term pricing, robust security features, and simplified access-control mechanisms. Notably, GCP enables rapid access to VMs using Secure Shell (SSH) keys, facilitating efficient code deployment and updates, in addition to providing backup redundancy for improved resilience.

The tool presents assessment results in two formats for clarity and usability:

- A tabular view showing results across individual security domains along with the overall maturity score.
- A gap analysis chart, offering a visual summary that simplifies reporting and highlights areas for improvement [121,122].

**(22) Health Information Systems Interoperability Maturity Toolkit (HISAMT)**

The HISAMT, developed by MEASURE Evaluation in collaboration with the Health Data Collaborative’s Digital Health and Interoperability Technical Working Group, is designed to help low-and middle-income countries systematically evaluate their capacity for HIS interoperability [123]. It includes the following:

1. A Maturity Model with defined domains and progression pathways for HIS interoperability.
2. An assessment tool that enables structured national or sub-national evaluations.
3. A users’ guide to support implementation and interpretation.

Since its initial release (v0.5 in 2017), the toolkit has evolved—with Version 1.0 launched in January 2019—incorporating lessons from adopters in Ghana and Uganda. The toolkit maps major HIS interoperability components across key domains—such as leadership, governance, workforce, and technology—creating a clear, stage-based Maturity Model. This allows countries to (a) assess their current maturity level, (b) identify gaps, and (c) plan progressive steps toward interoperable systems [124]. Available in English and French, the toolkit supports health ministries and implementing partners to perform the following:

- Benchmark interoperability maturity;
- Advocate for investment and policy change;
- Build roadmaps for digital transformation.

**(23) Digital Health Profile and Maturity Assessment Toolkit (DHPMAT)**

The Digital Health Profile and Maturity Assessment Tool (DHPMAT) was developed to support Pacific Island Countries (PICs) in leveraging digital technologies to advance national health priorities. Its creation drew upon insights from four existing maturity assessment frameworks that address the needs of health systems, organizations, and individual users: the Global Digital Health Index, Informatics Capability Maturity Model (ICMM), Health Information Systems Interoperability Maturity Toolkit, and Health Information System Stages of Continuous Improvement Toolkit [125].

The DHPMAT’s conceptual framework was particularly shaped by the WPRO eHealth Regional Action Agenda and the ICMM, providing a structured approach to assessing digital health maturity. The WHO Collaborating Centre on eHealth, WHO Western Pacific Regional Office (WPRO), and key stakeholders—including the WHO Department of Pacific Support, Pacific Health Information Network (PHIN), the Pacific Community, and WHO country office representatives—used a systematic co-creation approach to develop the tool [125]. This collaborative process included the following:

- Co-creating the Digital Health Profile (DHP);
- Defining both quantitative and qualitative country-specific indicators;
- Co-developing the Digital Health Maturity Assessment Tool (DHMAT);
- Aligning DHP indicators with the maturity of essential digital health foundations as assessed by the DHMAT;
- Iteratively refining and validating indicators and the DHP with input from key informants;
- Developing localized maturity assessment criteria;
- Finalizing the DHPMAT framework;
- Conducting multiple iterations of the DHPMAT to refine its content and structure;
- Testing the initial version with key informants, followed by further evaluation at a PHIN workshop.

The final DHPMAT framework comprises three key dimensions:

- Digital health foundations;
- Maturity assessment;
- Quality improvement.

Together, these dimensions help PICs systematically assess and strengthen their digital health environments, supporting both strategic planning and continuous improvement.

**(24) Information Systems for Health Standard Assessment Method (IS4HMM)**

The Information Systems for Health (IS4H) framework is designed to assess the maturity of Health Information Systems and the organizational capabilities required to operate, integrate, and benefit from them. Its primary purpose is to help assessors understand the application of IS4H within public health organizations and technological environments [126].

The IS4H framework is built upon a structured body of theories and conceptual models and is organized into five core components [126]:

Conceptual Framework:

- Acts as a conceptual bridge between guiding principles for health information systems and the strategic objectives of public health organizations. It ensures alignment between health information practices and broader public health goals.

Tactical Framework:

- Supports planning, risk identification, and prioritization of activities related to IS4H adoption. It addresses operational and cultural challenges, helping to balance local engagement and organizational support for health information systems.

Strategic Framework:

- Functions as a roadmap guiding health organizations toward their strategic goals within the IS4H program, outlining the vision, priorities, and actions needed for long-term success.

Concepts, Processes, Services, and Products:

- Defines key concepts within the health information systems domain and promotes shared understanding among health professionals. It also describes the processes necessary to deliver services and the benefits that they produce, providing a foundation for developing actionable plans based on IS4H best practices.

Trust-Based Framework:

- Provides tools and guidance for applying the IS4H Maturity Model Level Assessment, allowing organizations to appraise their current maturity level. Trust is positioned as a foundational element, supporting transparency and continuous improvement.

Learning Framework:

- Focuses on building capacity and fostering continuous learning within the IS4H environment. This component outlines learning objectives, processes, and tools to help health organizations and professionals adapt and evolve alongside advancements in Health Information Systems.

Together, these components form a comprehensive structure to help public health organizations assess their information systems’ maturity, guide strategic development, and foster a culture of trust and learning.

**(25) Infrastructure Adoption Model (INFRAM)**

The Infrastructure Adoption Model (INFRAM) is a structured framework developed by the Healthcare Information and Management Systems Society [127] to assess and enhance the maturity of a healthcare organization’s IT infrastructure. INFRAM provides a roadmap to help institutions align their network, compute, and data center capabilities with clinical and business objectives in a digitally transforming healthcare environment.

INFRAM evaluates five key domains:

1. Security—Ensuring protection of patient data and system integrity.
2. Mobility—Supporting clinicians’ access to systems and information anywhere, anytime.
3. Collaboration—Enabling communication and integration across systems and care teams.
4. Transport—Assessing the capacity and speed of networks and connectivity.
5. Data Center—Evaluating the resiliency and scalability of core IT systems.

The model follows eight progressive stages (Stage 0 to Stage 7), similar in structure to HIMSS’s EMRAM (Electronic Medical Record Adoption Model). Each stage defines a level of maturity, from basic network connectivity (Stage 0) to fully optimized infrastructure with predictive capabilities and real-time data analytics (Stage 7).

INFRAM is increasingly used by hospital CIOs, CTOs, and IT teams to ensure that infrastructure is not a limiting factor in digital transformation. The model emphasizes that robust and scalable IT infrastructure is essential for delivering high-quality, data-driven care [127].

### 5.3. Specialized Domain Models

**(26) The Telemedicine Service Maturity Model (TMSMM)**

The Telemedicine Service Maturity Model (TMSMM) was developed through an iterative process involving telemedicine professionals from five provincial health departments in South Africa’s Department of Health (DoH). The model was created to address the need for a structured framework to assess the maturity of both existing and planned telemedicine initiatives, enabling informed decision-making for the sustainable delivery of telemedicine services [102]. The TMSMM is structured around three key dimensions:

- eReadiness Categories;
- Stages of the Telemedicine Process;
- Maturity Levels.

The intersection of these dimensions forms a matrix, where each cell represents a specific aspect of telemedicine service maturity with distinct meaning and functionality. The model accounts for processes and factors operating at the micro, meso, and macro levels, encompassing the full range of activities required for successful telemedicine implementation.

The TMSMM’s maturity scale is adapted from the Capability Maturity Model (CMM), providing a set of generic maturity level indicators [102].

The model identifies six key determinants of telemedicine success [128]:

- Technology and Maintenance: Availability and reliability of ICT infrastructure, user training, and system usability.
- Policy and Legislation: The presence of supportive government and institutional policies, standardization efforts, and security measures.
- Individual Users: The trust and willingness of healthcare providers and decision-makers, evidence generation, and openness to process changes.
- Organizational Processes: Effective decision-making structures and streamlined work procedures.
- Planning and Financial Sustainability: Viable business models that ensure the long-term continuity of telemedicine services.
- Community Interaction and Involvement: Engagement with and involvement of the local community in telemedicine initiatives.

The TMSMM provides a comprehensive approach to measure, manage, and optimize all components of a telemedicine system, as well as the broader healthcare environment in which telemedicine is implemented [128].

**(27) Continuity of Care Maturity Model (CCMM)**

The Continuity of Care Maturity Model (CCMM) is a strategic framework developed by HIMSS to support healthcare organizations in implementing and advancing continuity of care practices. Its goal is to optimize health outcomes for both patients and the broader healthcare system by enabling seamless care coordination across various providers and care settings [129].

The CCMM defines a progressive eight-stage roadmap (Stages 0–7), outlining the key capabilities healthcare organizations must develop to enhance interoperability, information exchange, care coordination, patient engagement, and data-driven health management. The ultimate aim is to improve both individual and population health outcomes.

Organizations begin at Stage 0, where there is limited interoperability, and advance through the stages by first establishing basic peer-to-peer data exchange (Stages 1–3), where structured and discrete data formats begin to be used effectively.

In Stages 4–6, the focus shifts toward higher levels of coordinated care, incorporating wider participation from healthcare providers and patients.

At Stage 7, organizations achieve fully optimized, patient-centered healthcare delivery, characterized by interconnected, dynamic systems that support holistic, coordinated care across the continuum. 

CCMM Assessment Dimensions:

- EMR Functionality: Supports longitudinal patient tracking, referral processes, and comprehensive discharge summaries.
- Interoperability Standards: Implements open standards such as HL7, FHIR, and SNOMED CT to enable structured data exchange.
- Data Governance: Ensures role-based access controls, consent management, and audit trails to safeguard patient data.
- Care Pathway Alignment: Enables multidisciplinary, team-based care pathways within the EMR.
- System Integration: Achieves seamless communication and synchronization between core Health Information System (HIS) modules, such as laboratory, radiology, and pharmacy.
- Patient Engagement: Facilitates patient access to health records, digital follow-ups, and automated reminders to promote active participation in their care.
- The CCMM serves as both an assessment and a strategic planning tool, helping healthcare organizations benchmark their current capabilities and define the necessary steps toward integrated, patient-centered care delivery [129].

Morover, Kim and Namkoong [130] developed the Digital Health Communication Maturity Model, identifying key developmental stages in how healthcare organizations adopt and optimize digital communication strategies. Their systematic review emphasizes the importance of structured digital messaging, patient engagement, and provider communication pathways as essential enablers of modern healthcare delivery.

**(28) Interoperability Maturity Model (IMM)**

The Interoperability Maturity Model (IMM) provides a structured and strategic framework for assessing the capability of healthcare organizations to share and utilize health data across systems in a secure, standardized, and meaningful way. As healthcare delivery becomes increasingly data-driven, IMM frameworks have emerged as essential tools for guiding the development of interoperable health information infrastructures. The National eHealth Transition Authority [131] in Australia first formalized the IMM to help eHealth organizations assess and enhance their interoperability capabilities. NEHTA’s IMM defines an iterative, multi-stage process through which organizations can benchmark their current performance, set interoperability improvement goals, and align internal systems with national or regional eHealth frameworks. It focuses on both technical and organizational aspects, offering guidelines to evaluate semantic interoperability, data exchange standards, governance structures, and workflow alignment. Complementing this, Knight et al. [132], in a presentation delivered at the Pacific Northwest National Laboratory’s Interoperability Project Partners Meeting, proposed a U.S.-centric perspective on IMM. Knight emphasized the increasing necessity for cross-system integration, especially in contexts where patients receive care from multiple providers and systems. His model outlines a progressive maturity path, highlighting the evolution from basic point-to-point data exchange to full semantic interoperability and automated decision support integration. Knight’s IMM supports both a technical architecture evaluation and policy-based interoperability planning. Together, these IMM frameworks can evaluate a range of key domains:

- Governance and policy alignment;
- Use of open standards (e.g., HL7, FHIR, CDA, SNOMED CT);
- Technical infrastructure readiness;
- Organizational workflows and data lifecycle management;
- Security, privacy, and consent mechanisms;
- Monitoring and performance metrics.

These models serve as practical tools for healthcare organizations, governments, and IT strategists to benchmark their interoperability capabilities, align health IT systems, and design interventions that improve the continuity and quality of care. By leveraging IMM, stakeholders can not only track progress over time but also prioritize investments and facilitate multi-stakeholder collaboration in digital health transformation efforts.

**(29) Health Information Network Maturity Model (HIN)**

The Health Information Network (HIN) framework is one of several tools designed to help planners and operators translate best practices into actionable strategies. It enables a structured, objective assessment of an HIN’s operational capabilities and helps develop plans to enhance the level of service and value delivered [133].

The framework is structured around 10 capability domains:

- Vision and Engagement
- Governance
- Policy and Legislation
- Skills and Resources
- Funding
- Model Practice
- Success Metrics
- Clinical Use Cases
- Technology and Applications
- Security and Privacy

Each domain is assessed across five maturity levels:

- Initial
- Anticipate
- Interoperate
- Collaborate
- Optimize

These maturity levels describe the progressive behaviors, practices, processes, and capabilities that an HIN must develop over time. They also outline the key milestones an HIN needs to achieve to reliably and sustainably deliver the infrastructure and services required to support health outcomes and healthcare delivery across a province or territory. The HIN Maturity Model provides a comprehensive roadmap for strengthening network operations, promoting interoperability, and ensuring secure, effective health information exchange within and across healthcare systems [133].

**(30) Healthcare usability Maturity Model (UMM)**

Three widely accepted goals for usability are greater effectiveness, efficiency, and user satisfaction. In the context of electronic health records (EHRs), poor usability has been shown to negatively affect clinician productivity and may even contribute to medical errors [134]. Given that EHRs are often implemented to reduce errors and improve clinical outcomes, integrating usability principles into the development and deployment of these systems is critical. Recognizing this need, HIMSS initiated the development of a Usability Maturity Model (UMM) through its Usability Task Force, co-led by Staggers and Rodney [134]. The model was informed by an evaluation of the characteristics of three established Usability Maturity Models from other domains [135,136], adapting them specifically to healthcare. The UMM identifies key elements and milestones required to successfully embed usability into a healthcare organization’s processes [137]. It enables institutions to assess their current level of usability integration and provides guidance on how to advance to more mature stages. The model outlines five progressive phases [135]:

- Unrecognized—Lack of awareness or understanding of usability principles.
- Preliminary—Sporadic inclusion of usability practices, often inconsistent.
- Implemented—Usability is recognized as valuable and systematically considered.
- Integrated—Usability practices are embedded across all relevant workflows and departments.
- Strategic—Usability is treated as a strategic asset with allocated budgets and resources; outcomes are measured and used to drive organizational strategy.

**(31) Hospital Cooperation Maturity Model (HCMM)**

The Hospital Cooperation Maturity Model (HCMM) was developed to assess and improve both intra-organizational and inter-organizational cooperation within and among hospitals [138]. The model builds on the Maturity Model paradigm and is structured similarly to the Capability Maturity Model (CMM), though it represents a novel framework tailored specifically for healthcare collaboration. Key design principles are as follows:

- Opportunity: HCMM responds to the growing need for enhanced cooperation in healthcare, focusing on optimizing collaborative structures and workflows in hospital environments. It is a newly developed model rooted in contemporary healthcare challenges.
- Scope: The model is targeted, concentrating on strategic, organizational, and technical dimensions that influence cooperative efficiency. Its primary users are hospital decision-makers seeking to assess and improve collaboration.
- Maturity Concept: HCMM employs a multidimensional measurement framework. It evaluates both “as-is” (current state) and “to-be” (desired future state) maturity levels across strategic, organizational, and technical capabilities.
- Design Decisions: The model combines theory-based constructs with practical insights gathered from hospital environments. It aims to diagnose challenges and offer a roadmap for improvement across multiple dimensions of cooperation.
- Evaluation: The HCMM was evaluated using both ex-ante methods (e.g., structured questionnaires) and naturalistic settings involving real-world user feedback to validate both content and usability.

The HCMM consists of three distinct layers or dimensions:

1. Strategic Layer—Assesses the hospital’s capacity to establish and manage collaborations with external healthcare partners.
2. Organizational Layer—Focuses on internal cooperation across departments and units within the hospital.
3. Information Layer—Measures the adequacy and effectiveness of the hospital’s IT infrastructure to support internal and external collaboration.

**(32) Health Game Maturity Model (HGMM)**

The Health Game Maturity Model (HGMM) is a framework designed to help healthcare organizations assess and improve their use of gaming and gamification. Recognizing that gaming technologies can positively influence human behavior and enhance interaction, the HGMM assists organizations in understanding their current position in gamification adoption and in identifying the steps needed to progress to higher maturity levels [139].

In healthcare, gamification can range from simply making routine tasks more enjoyable to fully integrating gaming into clinical processes that improve health outcomes. Organizations at higher levels of maturity use gamification not only to engage staff or patients but also to drive measurable improvements in healthcare delivery and outcomes.

The HGMM follows a Capability Maturity Model (CMM)-based approach and evaluates an organization across four key perspectives, each with five levels of maturity:

1. Value Perspective: Focuses on the outcomes and benefits generated by gamification.

Non-existent: No use of games or perceived value.

Pleasure: Games are used for fun without targeted outcomes.

Passion: Games promote flow, engagement, and learning, supporting healthcare simulations.

Purpose: Games are integrated into healthcare-delivery processes, supporting innovation and treatment.

Healthcare Profit: Gamification improves healthcare quality and reduces costs.

2. Process Perspective: Assesses how well gamification is supported by organizational processes.

Ad hoc: Processes are informal, inconsistent, and reactive.

Repeatable: Gamification processes are repeatable but remain project-specific and reactive.

Defined: Organization-wide proactive gamification processes are established.

Managed: Processes are measured and systematically controlled.

Optimized: There is continuous monitoring and improvement of gamification processes.

3. Coverage Perspective: Reflects the breadth of gamification adoption across the organization.

None: No gamification present.

Individual: Gamification is used at the individual level.

Entity: Applied within specific departments or functional groups.

Institution: Adopted organization-wide across the entire healthcare institution.

Ecosystem: Fully integrated horizontally and vertically across the healthcare ecosystem, including external partners and users.

4. Type Perspective: Describes the nature and complexity of the gaming technologies used.

Off-line: Traditional offline games (e.g., board games).

Single-player: Online single-player gamification applications.

Multi-player: Online multi-player games supporting collaboration.

Group Playing: Online games designed for coordinated group participation.

Massively Multiplayer Online (MMO): Large-scale, networked multi-user gaming environments.

Healthcare organizations can use the HGMM to self-assess across these four perspectives, helping them align gamification efforts with their strategic goals and identify areas for growth. The model supports a structured approach to integrating gaming technologies in ways that advance patient care, workforce engagement, and overall health system performance.

**(33) Maturity Model of Hospital (MMH)**

The Maturity Model of Hospital (MMH), as proposed by Furukubo et al. [140] is a structured framework designed to guide hospitals through continuous improvement by assessing and enhancing their operational and organizational maturity. The MMH was developed to help hospitals (1) systematically assess their current capabilities, (2) identify areas for improvement, (3) create a roadmap for achieving higher levels of performance and quality, and (4) align organizational practices with best standards in healthcare delivery and management.

The Maturity Model of Hospital adopts a staged approach, inspired by models like the Capability Maturity Model (CMM) from software engineering. It defines five maturity levels, each representing a stage in the hospital’s evolution in terms of management, quality assurance, and patient care.

- Initial (Level 1): Processes are ad hoc, chaotic, or reactive; success depends on individual effort and a lack of standardization.
- Managed (Level 2): Basic project and process management is in place; repeatable procedures exist for specific areas and more consistent outcomes.
- Defined (Level 3): Organization-wide standards and policies are established; processes are documented and integrated and staff are trained in standardized procedures.
- Quantitatively Managed (Level 4): Performance metrics are collected and analyzed; data-driven decision making is emphasized and continuous monitoring and control.
- Optimizing (Level 5): Focus on continuous improvement and innovation; lessons learned are systematically incorporated with proactive problem prevention and process optimization.

**(34) High Reliability Health Care Maturity Model (HRHCM)**

Despite sustained and widespread efforts to improve healthcare quality, preventable patient harm remains a persistent challenge in many healthcare organizations [141]. One major obstacle has been the lack of a structured framework to categorize and assess hospitals based on their alignment with high reliability organization (HRO) principles. This gap has limited the ability of healthcare institutions to systematically implement and sustain evidence-based HRO practices. The High Reliability Health Care Maturity Model (HRHCM) was developed to address this need, offering a roadmap for organizations aiming to achieve zero patient harm. The model enables hospitals to identify and develop the organizational capabilities required for high reliability, emphasizing three foundational domains: Leadership, Safety Culture, and Robust Process Improvement [142]. By advancing maturity in these areas, healthcare providers can significantly reduce risks and improve outcomes for patients.

**(35) GWAC Interoperability Maturity Model (GIMM)**

The GridWise Interoperability Maturity Model (GIMM) was developed by the GridWise Architecture Council (GWAC) to help communities of organizations assess and improve their interoperability capabilities. Drawing inspiration from process-improvement frameworks such as SEI’s Capability Maturity Model Integration^®^ (CMMI^®^) and Australia’s National E-Health Transition Authority (NEHTA), the GIMM enables organizations to establish a baseline interoperability level and identify critical gaps and priorities for advancement [132].

The GIMM was designed alongside the Interoperability Roadmap Methodology, reflecting their close alignment. While GIMM assesses an organization’s current interoperability maturity, the roadmap methodology helps develop a strategic plan for advancing it. The ultimate aim is to increase interoperability maturity across a technology ecosystem in a way that balances technological possibilities, projected advancements, and the cost–benefit ratio of the effort.

As part of a broader technology integration strategy, the GIMM tool supports roadmap development for smart technology deployment and integration. The roadmap process engages entire ecosystems or communities of organizations, guiding them to define a shared vision of interoperability and develop prioritized actions to achieve it. These roadmaps are practical guides for collaborative progress in technology domains.

The GIMM framework defines 33 interoperability criteria, organized into six categories, providing a structured way to quantify interoperability maturity in technology integration efforts. It is specifically targeted at the following:

- Stakeholders working on standards, guidelines, and supporting resources to improve the integration of devices and systems, and
- Anyone seeking a deeper understanding of interoperability dimensions and the detailed structure of the Maturity Model.

In summary, GIMM is a strategic tool that helps technology-integration communities assess their current interoperability landscape, plan targeted improvements, and align technical efforts with the overall goals of the ecosystem [132].

**(36) Global Goods Maturity Model (GGMM)**

Digital Square, an initiative focused on coordinating global efforts to develop and share free, open-source digital health tools, has cataloged 18 mobile health (mHealth) solutions in its digital health software repository known as “global goods software” [143]. To support the advancement of these tools, Digital Square created the Global Good Maturity Model (GGMM), a framework designed to engage the digital health community in identifying priority areas for investment and improvement in global goods software [143].

The GGMM is particularly well-suited for evaluating the maturity of the Surveillance Outbreak Response Management and Analysis System (SORMAS) for several reasons:

- Health software focus in low-resource settings: GGMM emphasizes solutions intended for use in resource-constrained environments, aligning with SORMAS’s operational context.
- Aligned objectives: The goals of the GGMM closely match SORMAS’s mission of improving health surveillance and outbreak responses.
- Comparable scope: Many of the tools assessed through the GGMM framework share similar functionalities and use cases with SORMAS.

The GGMM 1.0 guideline defines a structured assessment approach, incorporating 15 sub-indicators organized under three core areas [144]:

- Global Utility;
- Community Support;
- Software Maturity.

The GGMM offers clear definitions for most indicators, making it a practical and accessible tool for self-assessment, although some indicators may require significant resources to be fully evaluated. While the model may benefit from some conceptual refinements to improve its applicability, it already serves as a valuable guide for driving software development toward greater global goods maturity, enhancing the scalability and sustainability of digital health interventions.

**(37) The Field Hospital Maturity Model (FHMM)**

The proposed Field Hospital Maturity Model (FHMM) is developed based on an extensive study of four core inputs: (1) the structural and organizational setup of field hospitals, (2) guideline and recommendation documents specific to field hospital deployment and operation, (3) related Maturity Models from similar domains, and (4) standards and regulations applicable to both affected countries and supporting nations. The resulting model defines a structured framework encompassing major developmental axes for field hospitals, as well as clearly defined maturity levels, allowing organizations to assess and position themselves along each axis [145]. The model is iterative, and ongoing refinements and validations are performed to enhance its accuracy and applicability. The FHMM comprises a global framework subdivided into three main axes, each supported by corresponding local models:

- Governance—This axis addresses the decision-making and command structure of field hospitals throughout their entire mission lifecycle, including situation assessment, deployment, operational phases, and withdrawal.
- Logistics—Encompasses the provision, storage, transport, and post-use collection of all resources (both medical and non-medical) necessary for field hospital functioning. These logistic operations may be internally managed or supported by external entities.
- Care—Refers to the clinical and medical services delivered during the field hospital’s operation phase.

Each of these axes includes a set of critical components and is assessed across five maturity levels:

- Unconsidered/Unknown—The axis is either poorly addressed or entirely neglected.
- Initial—The axis is recognized but addressed in an unstructured or ad hoc manner.
- Practiced—Formal processes are in place, but there is no consistent monitoring or assessment.
- Managed—The axis is governed by international standards and established procedures, with appropriate monitoring and management mechanisms.
- Improved—Processes are continuously optimized, with the systematic incorporation of changes and updates to standards and procedures.

This model provides a structured approach to evaluating and guiding the development and optimization of field hospitals in diverse and often challenging operational contexts.

**(38) Interoperability Maturity Assessment of a Public Service (IMAPS)**

The Interoperability Maturity Assessment of a Public Service (IMAPS) is a framework developed to evaluate and enhance the behavioral interoperability of digital public services. Its primary objective is to offer actionable insights into how these services can improve their interoperability maturity [146]. Upon completing an online assessment, users receive a report with tailored recommendations aimed at advancing their maturity level. IMAPS applies five core principles to generate its recommendations:

- Differentiation of Maturity Levels: Each semantic interoperability attribute distinguishes between at least two levels of maturity.
- Gradual Improvement Guidance: Recommendations are provided using improvement tables that guide services incrementally through maturity stages.
- Targeted Recommendations: If a service has not yet reached the highest maturity level for an attribute, IMAPS offers specific advice to reach the next level.
- No Redundant Advice: If the highest maturity level has already been achieved, no further recommendation is given for that attribute.
- Sliding Scale for Generic Improvements: Where maturity is measured on a continuous scale rather than discrete levels, general advice is provided to promote further improvement.

Each recommendation is linked to four elements: the specific question assessed, the current maturity level, the next level to be attained, and the practical advice to close that gap [146].

**(39) Health Information System Stages of Continuous Improvement Toolkit (SOCI)**

The Standards and Tools for Health Information Systems (SOCI) Toolkit was developed to address a foundational question in Health Information System (HIS) development: “What are the critical factors for HIS development and continuous improvement?” As a Maturity Model, the SOCI Toolkit delineates the progressive stages through which HISs can evolve to achieve higher levels of capability, functionality, and performance. It is specifically tailored to support continuous HIS-improvement efforts in low- and middle-income countries (LMICs). The SOCI Toolkit serves multiple essential purposes [147]:

- It provides a systematic measurement framework to describe and evaluate HIS components;
- It helps users and stakeholders set strategic goals for HIS advancement;
- It guides the development of improvement plans, enabling systems to move progressively toward higher levels of maturity.

The model outlines five stages of HIS maturity: emerging/Ad hoc, repeatable, defined, managed, and optimized. These stages are assessed across five core HIS domains, which are further broken down into 13 components and 39 subcomponents. Through this structure, the SOCI Toolkit enables health systems to benchmark their current status, identify gaps, and design targeted interventions to strengthen HIS functionality and performance over time.

**(40) Community Care Outcomes Maturity Model (C-COMM)**

The Community Care Outcomes Maturity Model (C-COMM) is a strategic framework developed by the Healthcare Information and Management Systems Society [148] to assess and advance the digital maturity of non-acute, community-based healthcare settings. These include services such as primary care, mental health, maternal and child wellness, and virtual care. C-COMM aims to build a person-centered, digitally enabled community care ecosystem by focusing on the following objectives: (1) Measure Digital Maturity: Assess the adoption and effective use of digital technologies across non-acute healthcare providers; (2) Enhance Patient Engagement: Use digital tools to identify gaps in access and improve connections between patients and their care teams; (3) Establish Governance: Promote clear policies and procedures to ensure consistent, secure, and coordinated care. C-COMM evaluates healthcare organizations across progressive stages of maturity, like other HIMSS models like EMRAM and INFRAM. It emphasizes the integration of technology, data, and workflows across care environments that extend beyond traditional hospitals. The model encourages the use of digital health tools that support longitudinal care, especially for patients with chronic conditions or complex needs. C-COMM enables digital transformation in community care through a structured roadmap, supports alignment with national health priorities and value-based care models, and helps care providers improve coordination, outcomes, and assessments, especially in underserved populations [148].

**(41) Integrated Care Maturity Model (IC-MM)**

The Integrated Care Maturity Model (IC-MM) is a framework developed to evaluate and guide the development of integrated care systems. It is particularly aimed at helping health systems assess their readiness and capability to deliver coordinated, patient-centered care across organizational and sectoral boundaries. The IC-MM has been notably developed and used by the European Innovation Partnership on Active and Healthy Ageing (EIP on AHA) and the Blueprint on Digital Transformation of Health and Care [149]. It is designed to support regions and countries in implementing integrated care for complex and chronic patients. The IC-MM typically includes 12 dimensions, each reflecting a key enabler for integrated care:

1. Readiness to Change—Organizational willingness and leadership support.
2. Structure and Governance—Policies and partnerships to support integration.
3. Information and eHealth Services—Use of digital tools for communication and data sharing.
4. Standardization and Simplification—Use of common protocols and processes.
5. Funding Models—Sustainable financing mechanisms for integrated care.
6. Incentives and Motivation—Professional and system-level incentives.
7. Process Coordination—Alignment of care pathways across providers.
8. Population Approach—Stratification and targeting based on patient needs.
9. Citizen Empowerment—Involving patients in care decisions and self-management.
10. Evaluation Methods—Measurement of outcomes and performance.
11. Breadth of Ambition—Scope of integration (local vs. system-wide).
12. Innovation Management—Capacity to adopt and scale new models.

Each dimension is typically assessed on a 5-point scale and intended to (1) help regions and health systems benchmark their current state; (2) support strategic planning for scaling integrated care; (3) encourage cross-sector collaboration, especially between health, social, and community services; and (4) promote value-based care, especially for aging and multi-morbid populations [146].

**(42) Collaboration Maturity Model (CollabMM)**

The Collaboration Maturity Model (CollabMM)—also referred to as the Collaborative Maturity Model in some literature—is a structured framework developed to evaluate and enhance an organization’s collaborative capabilities. First introduced in the early 2000s, the model was created to address the growing importance of explicit, measurable, and strategic collaboration within and across organizations [150]. CollabMM is inspired by traditional Maturity Models like the Capability Maturity Model (CMM) and CMMI and follows a multi-level progression structure, typically spanning from ad hoc collaboration to fully integrated and optimized collaboration practices. The model evaluates several key dimensions:

- Process Integration—The extent to which collaboration is embedded in formal business workflows.
- Governance and Roles—Definition of responsibilities, facilitation structures, and leadership mechanisms that support collaborative work.
- Supporting Tools—The use and integration of digital platforms and technologies (e.g., communication tools, shared documentation systems) that enable effective collaboration.
- Measurement and Improvement—The presence of metrics and feedback loops to assess collaborative performance and guide continuous enhancement.

Benefits of CollabMM:

- Visibility and Clarity: CollabMM transforms informal collaboration into a formalized, measurable process. This visibility supports better alignment with strategic objectives.
- Benchmarking: The model facilitates both internal and external benchmarking of collaborative practices, providing a reference point for organizational development.
- Targeted Improvements: By highlighting maturity gaps, CollabMM offers a roadmap for systematic improvement across tools, communication practices, governance, and team culture.
- Sector Versatility: CollabMM is applicable across a wide range of sectors, including healthcare, education, software development, and public administration, making it a flexible and scalable tool for organizational development.

Although often referenced in the context of enterprise collaboration and knowledge management, CollabMM is gaining traction in healthcare environments, particularly in projects requiring multi-disciplinary teamwork, cross-sector partnerships, or the integration of digital collaboration platforms.

### 5.4. Data and Analytics Models

**(43) Informatics Capability Maturity Model (ICMM)**

- The Informatics Capability Maturity Model (ICMM) is a framework designed to assess an organization’s ability to effectively collect, manage, and share health data, implement ICT solutions, ensure robust data governance, and leverage health business intelligence for integrated, multidisciplinary care delivery [151]. The ICMM aligns with broader informatics maturity frameworks and has been shown, in integrated primary care settings, to correlate with improved care coordination and health outcomes.

Maturity Levels [136]:

- Basic: Health IT systems are fragmented, unreliable, and lack coordination.
- Controlled: Systems provide consistent functionality, but data and expertise remain siloed.
- Standardized: Common standards and protocols enable broader data sharing and collaboration.
- Optimized: Processes are streamlined, efficient, and governed by formal policies.
- Innovative: Informatics capabilities drive continuous innovation and are embedded across the enterprise.

Key Dimensions of the ICMM:

- ICM1: Data collection, integration, and management (including HIS and EHR systems).
- ICM2: Information sharing and interoperability across healthcare districts and networks.
- ICM3: ICT-implementation practices and change management strategies.
- ICM4: Data quality management and governance frameworks for secure and reliable data handling.
- ICM5: Use of health business intelligence to drive improvements in population health outcomes.

Leadership Objectives Encouraged by the ICMM:

- Recognize informatics as a strategic asset that supports broader business and care-delivery objectives.
- Align IT investments with organizational goals and care-delivery strategies.
- View IT not merely as a support function but as a key driver of healthcare transformation.
- Implement IT-enabled change management practices that enhance organizational efficiency and clinical outcomes.

The ICMM serves as a roadmap for healthcare leaders to strengthen their informatics capabilities, helping organizations advance from basic functionality to enterprise-wide innovation.

**(44) The Healthcare Analytics Adoption Model (HAAM)**

The Health Catalyst developed the Healthcare Analytics Adoption Model (HAAM) as a structured framework for measuring the maturity of data warehouse utilization and analytics within healthcare organizations, analogous to the HIMSS Analytics EMRAM model. The initial phase of data collection, driven by rapid electronic medical record (EMR) deployment, does not inherently yield significant improvements in healthcare quality or cost [152]. HAAM emphasizes the importance of a foundational understanding in analytics before organizations progress to more advanced stages. It outlines a staged pathway for adopting analytics, expanding across four critical dimensions: (1) New Data Sources—Increasing the breadth and variety of data inputs; (2) Complexity—Employing progressively sophisticated analytical methods and data integration; (3) Data Literacy—Enhancing staff proficiency in interpreting and applying data insights; and (4) Data Timeliness—Improving the speed at which data informs decision-making, thereby reducing response cycles.

HAAM consists of nine levels of analytics maturity [152]:

Level 0: Point Solutions—Data exists in isolated, siloed systems with minimal integration.

Level 1: Enterprise Data Warehouse—A centralized repository is created, aggregating data across systems for enterprise-wide use.

Level 2: Standardized Vocabulary and Patient Records—Adoption of standardized clinical vocabularies (e.g., SNOMED, LOINC) and consistent patient record structures to enable interoperability and accurate data exchange.

Level 3: Automated Internal Reporting—Routine performance metrics and clinical indicators are automatically generated for internal management and quality improvement.

Level 4: Automated External Reporting—Automated processes support mandatory external reporting to regulatory bodies, payers, and public health agencies.

Level 5: Waste and Care Variability Reduction—Analytics are used to identify and reduce unnecessary variation in clinical practices and operational inefficiencies.

Level 6: Population Health Management with Suggestive Analytics—Analytics support proactive management of patient populations, using data-driven suggestions to optimize care and health outcomes.

Level 7: Clinical Risk Intervention with Predictive Analytics—Predictive models identify patients at risk for adverse outcomes, enabling targeted interventions and risk management.

Level 8: Personalized Medicine with Prescriptive Analytics—The most advanced level, where prescriptive analytics drive personalized care plans based on individual patient characteristics, genomic data, and predictive insights.

HAAM serves as a roadmap for healthcare organizations aiming to advance their analytics capabilities and leverage data more effectively to improve clinical, operational, and financial outcomes.

**(45) Business Intelligence Maturity Model (BIMM)**

Healthcare decision-makers today are confronted with growing demands for timely and accurate clinical and administrative information to support complex decision-making [153]. The application of Business Intelligence (BI) in healthcare is particularly challenging due to the integration of diverse data types and the multifaceted nature of the sector. Three key factors contribute to the complexity of implementing BI solutions in healthcare [153]:

- The need to integrate both clinical and financial data, which traditionally exist in siloed systems;
- The heterogeneity of data formats required to support high-level analytical functions;
- Increasing expectations from external stakeholders for reliable clinical and financial decision support.

To address these challenges, Brooks et al. [154] proposed a BI Maturity Model specifically tailored to healthcare organizations. This model emerged from a comprehensive literature review of existing BI maturity frameworks, critical success factors for BI, and the unique attributes of Healthcare Information Systems. The model is designed to be practical and actionable for healthcare management teams, providing the following core functionalities:

- A conceptual framework for managing BI deployment in healthcare environments;
- A focus on both operational/financial and clinical information requirements;
- Inclusion of key BI processes that are specific to healthcare, such as patient care pathways, regulatory compliance, and cost control;
- Integration of the people–process–technology triad to ensure a holistic maturity evaluation;
- Emphasis on quality dimensions, including system quality, information quality, and service quality;
- Clarity on the interrelationship between maturity levels and critical BI processes, grounded in theoretical and empirical foundations [154].

Building on this conceptual foundation, Gastaldi et al. [155] presented a multidimensional BI Maturity Model that maps maturity across four levels and three functional domains: functional, technological, diffusional, and organizational (Table 3).

**(46) Healthcare Data Quality Maturity Model (HDQM2)**

Data quality (DQ) is a critical factor in the development and operation of health systems, impacting both the quality of care delivery and the formulation of effective public policies [156]. Substantial evidence highlights that Health Information Systems often contain inaccuracies and data inconsistencies in patient records, which, if not addressed, may lead to adverse outcomes in healthcare services.

To address this, the Health Data Quality Maturity Model (HDQM2) was developed. This model builds upon established frameworks found in the literature but has been adapted to reflect the specific characteristics and needs of the health sector, particularly within local contexts.

HDQM2 conceptualizes a Health Information System through four foundational components: People, Processes, Data and Technology

The model is structured around the following key elements:

- Maturity Levels—defining the stages of advancement in data quality practices.
- Practices—standardized methods for improving data quality.
- Process Areas—aligned with phases of the data lifecycle (collection, storage, processing, analysis, and reporting).
- Value Creation—focusing on how data quality improvements contribute to better health outcomes and decision-making.

Core data quality dimensions addressed in the model include Accuracy/Correctness, Completeness, Uniqueness and Duplication Management.

By aligning these elements, HDQM2 provides a practical and context-aware roadmap for health organizations to evaluate and enhance their data quality practices systematically.

**(47) Data Quality Maturity Model (DDMM)**

The model will provide a framework for institutions to self-evaluate, report their status on several key items related to the quality of the real-world data (RWD) institutions, and make investments to advance their data systems. The Data Quality committee proposed a Maturity Model with a goal to describe a framework for assessing and advancing the competencies of an organization within a domain (e.g., healthcare technologies).

The DDMM proposes the following five stages of increasingly advanced and integrated levels of performance for healthcare systems with respect to data management [157].

- Stage 1. Conceptual: clinical processes capture data primarily in verbose documents, not as data; lack of organizational awareness of data utility, no effort to systematically manage healthcare data, lack of consistent or centralized governance, policies, and/or resources, data not organized centrally.
- Stage 2. Reactive: the enterprise can react to requests for analysis and respond to research requests but mostly accomplished by manual chart review and abstraction; data management inefficient and expensive, with only sporadic recognition of data utility beyond immediate use.
- Stage 3. Structured: clinical systems manage transactional data types (e.g., orders, transactions, laboratory results, medication prescriptions) as discrete data; support from leadership for centralized data governance and management of these data types at the enterprise level.
- Stage 4. Complete: granular and complete clinical data based on standardized clinical common data elements captured in the processes of care, integrated into those care processes; health systems data routinely and systematically represent data externally via various CDMs, including efficient queries, support for large number of research projects.
- Stage 5. Advanced: data linkage and aggregation across systems enabled and open to external queries; interoperability of clinical data enabled; multiple sources of sustainable funding support for research; engagement of regulatory and industry enterprises with enterprise data.

**(48) Adoption Model for Analytics Maturity (AMAM)**

The HIMSS Adoption Model for Analytics Maturity (AMAM) is a globally recognized framework designed to evaluate and improve healthcare analytics capabilities—ranging from foundational data governance to advanced predictive and prescriptive analytics [158].

AMAM is delivered across eight progressive stages (0–7), each representing a deeper and more integrated use of analytics to drive clinical, operational, financial, and strategic outcomes:

Stage 0—Fragmented Point Solutions: Basic, disjointed analytics tools with limited impact on decision-making.

Stage 1—Foundation Building: Data aggregation begins, and initial governance structures emerge.

Stage 2—Core Data Warehouse and Competency Center: Centralized data repositories with dedicated analytics teams.

Stage 3—Consistent Internal and External Reporting: Streamlined, reliable reporting across the organization.

Stage 4—Evidence-Based Care and Waste Reduction: Analytics support clinical decision-making and reduce variability.

Stage 5—Population Health and Economics Understanding: Analytics inform population health and cost management.

Stage 6—Clinical Risk Intervention and Predictive Analytics: Advanced predictive models are embedded in workflows.

Stage 7—Personalized Medicine and Prescriptive Analytics: Sophisticated analytics enable prescriptive, individualized care.

The model highlights four critical dimensions—data sources, analytic complexity, data literacy, and timeliness of insights—as organizations progress in their analytics maturity

### 5.5. Policy-Oriented Models

**(49) The Maturity Model for Health in All Policies (MMHiAP)**

The Maturity Model for Health in All Policies (MMHiAP) was developed to evaluate how effectively local governments implement policies aimed at addressing health inequalities. It builds upon the structure of the Capability Maturity Model (CMM) and is specifically tailored for assessing the implementation processes of Health in All Policies (HiAP) initiatives at the municipal level [159]. The MMHiAP consists of six hierarchical maturity levels, each defined by a set of 14 key characteristics that organizations must meet to progress to higher levels. The model assumes a sequential progression, meaning that an organization must fulfill all key characteristics of a given level before advancing to the next [160].

To determine an organization’s maturity level, the MMHiAP employs a triangulated assessment methodology, combining the analysis of health policy documents, online questionnaires, and semi-structured individual interviews [159]. This triangulation is essential to overcome the inherent challenges of evaluating HiAP implementation, such as varying interpretations of policy impact and institutional commitment. However, due to resource constraints, some studies applying the MMHiAP—such as the one referenced—have limited their data collection to questionnaires and interviews only.

**(50) Global Digital Health Index (GDHI)**

The Global Digital Health Index (GDHI) is an interactive digital platform designed to track, monitor, and evaluate the adoption and maturity of digital health technologies across countries. Functioning as both a benchmarking tool and a Maturity Model, the GDHI enables nations to assess their progress in digital health and compare their performance against global peers [161].

Developed using the WHO/ITU eHealth Strategy Toolkit [162] and informed by the WHO Global Observatory for eHealth Survey, the GDHI offers a standardized set of metrics to evaluate critical enablers of digital health. These include governance structures, interoperability frameworks, privacy and security policies, workforce capacity, and infrastructure readiness.

Key Objectives of the GDHI [161]:

- Benchmark digital health maturity and improve the quality of digital health systems at the national level.
- Track progress toward comprehensive and integrated digital health ecosystems.
- Identify gaps in funding and technical assistance, both within individual countries and across regions.
- Promote alignment among policymakers, donors, and implementers, following the Principles for Digital Development and the Donor Alignment for Digital Health framework.
- Highlight investment risks at the country level, providing greater transparency for funders and stakeholders.

The GDHI is currently undergoing a participatory redesign process, aimed at refining the index, updating its metrics, and integrating complementary digital health tools. This process is intended to ensure that the GDHI remains a practical and effective resource for countries, supporting assessment strengthening and the continuous monitoring of their digital health systems and broader digital health ecosystems.

## 6. Discussion

### 6.1. Definitions and Characteristics of Maturity Models

Maturity models (MMs) are structured frameworks that assess organizational capabilities across defined stages, often representing a progression from ad hoc to optimized states. While models like the Capability Maturity Model Integration for Services (CMMI) and the Business Process Orientation Maturity Model (BPOMM) originated in other sectors, they have been widely adopted or adapted to healthcare due to their emphasis on process structure, improvement, and alignment. These models typically include phased stages and diagnostic criteria, offering organizations a roadmap for progressive development. Despite their widespread use, MMs have drawn criticism for being static and overly generic, sometimes lacking contextual adaptability or empirical validation in specific domains [55,125]. Nonetheless, their popularity persists due to the clear benchmarking and performance-improvement pathways that they provide.

### 6.2. Applications in Healthcare Domains

This review identified 50 Maturity Models applied to healthcare and digital health ecosystems. These were categorized into five types: process-oriented, technology-focused, specialized domain, data and analytics, and policy-oriented models.

Process-oriented models, such as CMMI and BPOMM, focus on internal workflows, service delivery, and process improvement. Technology-focused models (e.g., EMRAM, HISMM, HCSMAF) evaluate the adoption and integration of digital systems, clinical applications, cybersecurity, and infrastructure maturity. Specialized domain models like the PACS Maturity Model and Telemedicine Service Maturity Model (TMSMM) target narrower domains such as imaging and remote care, offering high specificity.

Data and analytics models (e.g., ICMM, HAAM) reflect a shift toward data-driven decision-making. These frameworks assess how well organizations collect, analyze, and leverage data for clinical and operational improvement. Finally, policy-oriented models (e.g., MMHiAP, GDHI) extend assessments to national or cross-institutional governance, evaluating the maturity of public health policy environments and regulatory strategies.

This variety shows a move from isolated process assessments to more integrated, multidimensional frameworks that combine clinical, technological, and governance dimensions (Table 4).

While many models share common themes such as staged maturity progression and multidimensional assessments, they differ significantly in their applicability to healthcare contexts, scope of assessment, and practical implementation guidelines. This reinforces the importance of contextualizing model selection and application to meet the specific needs of each healthcare organization.

Although this review compares Maturity Models by domain, structure, and focus, a formal benchmarking framework remains challenging due to inconsistent reporting and limited empirical validation across studies. Nevertheless, several key dimensions could support future comparative evaluations, including the following: (1) ease of implementation, (2) level of empirical validation, (3) contextual adaptability, (4) stakeholder usability, and (5) market or institutional adoption. Developing a standardized set of evaluation criteria would enhance the comparative rigor and guide healthcare organizations in selecting the most appropriate Maturity Model for their needs. However, such benchmarking efforts require access to implementation data and collaboration among researchers, providers, and system vendors.

### 6.3. Contextualization in Healthcare Environments

While the Maturity Models identified in this review provide structured frameworks for assessment and improvement, their application in healthcare environments requires careful contextual adaptation, especially for models originally designed for IT or industrial sectors.

#### 6.3.1. Public vs. Private Hospitals

Private hospitals typically benefit from greater financial and operational flexibility, enabling them to adopt more advanced models such as INFRAM or HIMSS EMRAM, which require significant investments in technology and organizational change. Public hospitals, on the other hand, often face budgetary constraints and regulatory complexities, making it more challenging to fully implement comprehensive models. In these contexts, more scalable and accessible models, such as the Healthcare IT Maturity Model (HIT-MM) or the Health Information Systems Interoperability Maturity Toolkit (HISAMT), may be more practical starting points.

#### 6.3.2. Developed vs. Emerging Economies

In developed countries, the adoption of sophisticated Maturity Models is supported by robust infrastructures, stable regulatory environments, and a highly skilled workforce. Models such as INFRAM, HAAM, and the Global Digital Health Index (GDHI) are well-suited to these environments. In contrast, emerging economies face challenges such as limited resources, a fragmented infrastructure, and gaps in the workforce capacity. For these settings, simpler, modular models like the eHMM and GPIMM offer a more realistic approach to gradually advancing digital health maturity.

#### 6.3.3. Primary vs. Tertiary Care Settings

In primary care environments, where the focus is on preventive care and basic health services, simpler models like the General Practice Information Maturity Model (GPIMM) or the Continuity of Care Maturity Model (CCMM) are more appropriate. These models help assess and improve foundational processes without the complexity of tertiary care systems. In contrast, tertiary care hospitals, where clinical and technological processes are more complex, benefit from models such as the PACS Maturity Model (PMM), the Hospital Information System Maturity Model (HISMM), and the High Reliability Health Care Maturity Model (HRHCM), which address clinical quality, patient safety, and integrated IT infrastructure.

#### 6.3.4. Limitations of Adapted Models from Other Sectors

A notable limitation in the field is the adaptation of Maturity Models originally designed for IT or industrial environments, such as the Capability Maturity Model Integration (CMMI) and the Cloud Security Capability Maturity Model (CSCMM). While these models provide robust process-improvement frameworks, they may lack sensitivity to the unique characteristics of healthcare, such as the following:

- The criticality of timely clinical decision-making;
- The ethical and regulatory sensitivities surrounding personal health data;
- The highly interdisciplinary nature of healthcare teams.

Therefore, applying these models in healthcare requires contextual adaptation and empirical validation, ensuring that clinical safety and patient-centered care remain the primary focus over purely operational efficiency. Therefore, applying these models in healthcare requires contextual adaptation and empirical validation, ensuring that clinical safety and patient-centered care remain the primary focus over purely operational efficiency. For instance, CMMI does not include metrics related to patient outcomes, interoperability with standards like HL7 or FHIR, or compliance with health-specific regulations such as HIPAA. Moreover, these adapted models are often validated in industrial settings but lack peer-reviewed evidence of their effectiveness in clinical or hospital environments, limiting their scientific reliability in healthcare maturity assessments.

### 6.4. Practical Implications and Model Selection Guidance

Given the wide variety of Maturity Models identified in this review, healthcare organizations may face challenges in selecting the most appropriate one for their specific needs. The choice of a Maturity Model should be guided by the organization’s current level of digital maturity, strategic goals, resource availability, and operational priorities.

Organizations with low digital maturity (e.g., limited electronic medical record use or fragmented IT infrastructure) may benefit from adopting foundational models such as the HIMSS Analytics EMRAM, which provides a structured roadmap for electronic medical record adoption and clinical process digitization.

Healthcare providers seeking to enhance infrastructure capabilities should consider models like the Infrastructure Adoption Model (INFRAM), which focus on IT architecture, cybersecurity, and mobility—critical elements for supporting advanced Health Information Systems.

Primary care and general practice settings may find the General Practice Information Maturity Model (GPIMM) valuable for assessing their transition from paper-based to electronic record systems and for planning staff training and process improvements.

Organizations aiming for interoperability and health information exchange should prioritize models such as the Interoperability Maturity Model (IMM) or the Health Information Systems Interoperability Maturity Toolkit (HISAMT), which provide clear frameworks for achieving semantic and technical interoperability.

Hospitals with advanced HIS adoption might focus on specialized domains like PACS Maturity Model (PMM) for imaging, Healthcare Cloud Security Maturity Assessment Framework (HCSMAF) for security, or the High Reliability Health Care Maturity Model (HRHCM) for fostering a safety culture and robust process improvement.

Public health agencies and policy bodies can leverage models such as the Maturity Model for Health in All Policies (MMHiAP) or the Global Digital Health Index (GDHI) to assess and advance systemic digital health capabilities at national or regional levels.

No single model fits all healthcare contexts. Organizations are advised to conduct a preliminary self-assessment to identify key gaps and then select a model (or a combination of models) that aligns with their improvement priorities and capacity for change.

### 6.5. Limitations of the Review

Despite providing a comprehensive overview of healthcare Maturity Models, this review is subject to several limitations that should be considered when interpreting its findings.

#### 6.5.1. Scope of the Literature Search

The search strategy primarily focused on academic databases and grey literature from well-known health and IT organizations. However, some emerging Maturity Models or unpublished frameworks from smaller healthcare systems, consultancy practices, or regional health authorities may not have been captured. As a result, this review may not fully represent the entire global landscape of healthcare maturity assessments.

#### 6.5.2. Heterogeneity of Model Definitions

There is significant variability in how Maturity Models are defined, structured, and applied across studies and sectors. Some models are highly detailed with validated metrics, while others remain conceptual frameworks lacking empirical testing. This heterogeneity limited the ability to directly compare the effectiveness, scope, and validation status of the models.

#### 6.5.3. Limited Empirical Validation

Several of the models identified, especially those adapted from other industries (e.g., IT, business process management), have limited empirical validation in healthcare environments. This raises concerns about their practical applicability and effectiveness when implemented in clinical settings, especially in diverse health system contexts.

#### 6.5.4. Geographical and Sectoral Bias

Most models were developed and validated in high-income countries with advanced digital health infrastructures, such as the United States, Europe, and Australia. Consequently, the applicability of these models to low- and middle-income countries (LMICs) or to resource-constrained healthcare settings may be limited without significant contextual adaptation.

#### 6.5.5. Focus on Model Characteristics over Outcomes

This review emphasized the characteristics and structures of the Maturity Models rather than their impact on healthcare outcomes. Future studies should investigate how the adoption of these models correlates with measurable improvements in clinical quality, patient safety, operational efficiency, and digital transformation.

#### 6.5.6. Limited Coverage of Commercial Benchmarking Models

While this review focused primarily on peer-reviewed and academically documented Maturity Models, it did not systematically analyze proprietary or commercial models such as HIMSS EMRAM, INFRAM, or IDC Health IT Maturity Models. These commercially available frameworks are widely used by hospitals and healthcare providers and typically rely on benchmark-driven scoring, certification pathways, and structured implementation stages. However, they are often less transparent in their development methods and may prioritize operational or financial outcomes over scientific rigor or clinical impact. Future reviews could more fully compare these market-oriented models to academic frameworks to evaluate their relative strengths and evidence base.

### 6.6. Recommendations for Future Research

This review highlights several opportunities for further investigation to advance the development and application of Maturity Models in healthcare:

#### 6.6.1. Empirical Validation in Diverse Healthcare Settings

Many Maturity Models identified in this review lack rigorous validation in real-world healthcare environments, particularly in low-resource and public health settings. Future studies should conduct empirical case studies, longitudinal evaluations, and pilot implementations to assess the practical effectiveness of these models across different healthcare contexts.

#### 6.6.2. Development of Healthcare-Specific Models

Several models reviewed were originally developed for generic IT or industrial sectors and later adapted for healthcare. There is a need to design healthcare-specific Maturity Models that incorporate clinical workflows, patient safety, ethical considerations, and regulatory compliance from the outset, rather than adapting pre-existing industrial frameworks.

#### 6.6.3. Comparative Studies of Model Effectiveness

Little research has compared the effectiveness of different Maturity Models in improving healthcare outcomes. Future work should explore comparative analyses, evaluating which models provide the most value for specific types of healthcare organizations, such as hospitals versus primary care clinics or private versus public sector entities.

#### 6.6.4. Integration Across Domains

Many models focus narrowly on a single domain (e.g., IT infrastructure, process management, or interoperability). Future research should investigate the development of integrated Maturity Models that address technology, process, governance, clinical care, and patient engagement in a holistic framework.

#### 6.6.5. Adaptation for Emerging Technologies and Challenges

As healthcare organizations increasingly adopt artificial intelligence (AI), cloud computing, telemedicine, and real-time data analytics, Maturity Models must evolve to assess these emerging capabilities. In particular, there is a growing need for models that explicitly incorporate AI-driven decision support systems and the Fast Healthcare Interoperability Resources (FHIR) standard, which enables efficient and secure health data exchange. These technologies are reshaping Health Information Systems, and future Maturity Models should evaluate readiness not only for technical implementation but also for ethical, legal, and workflow integration. Incorporating these dimensions would ensure that maturity assessments remain aligned with current and future clinical innovations.

#### 6.6.6. Tools and Automated Assessments

Few models provide practical assessment tools, dashboards, or automated platforms to assist organizations in applying the frameworks. Future research could develop digital assessment tools to improve usability and enable continuous maturity tracking.

### 6.7. Summary of Identified Maturity Models

The healthcare sector has adopted a wide range of Maturity Models to assess and guide the development of its digital, organizational, and clinical capabilities. Table 5 provides a comprehensive summary of the Maturity Models identified in this review, outlining their health focus, key dimensions or factors assessed, and their original authors. These models reflect the multidisciplinary nature of healthcare maturity assessments, covering domains such as process management, information systems, cybersecurity, interoperability, and patient care. By consolidating this information, Table 5 offers a structured overview that can assist healthcare organizations, policymakers, and researchers in selecting or adapting Maturity Models that align with their strategic goals and operational needs.

The adoption of Maturity Models in healthcare is particularly relevant given the persistent challenges faced in HIS implementation—such as resistance to change, lack of interoperability, and inadequate contextual adaptation. By providing structured frameworks for assessment and gradual improvement, Maturity Models can help organizations systematically address these barriers, prioritize resource allocation, and guide strategic planning aligned with digital health objectives.

## 7. Conclusions

Many hospitals and healthcare systems are adopting delivery-system reform to better align provider incentives with the goals of enhancing the patient care experience, improving population health outcomes, and reducing per capita healthcare costs. In this context, the role of Health Information Systems (HISs) becomes increasingly critical. These systems manage vast amounts of administrative and clinical data across numerous patients, but the true value lies not merely in data access, but in transforming this data into actionable insights that enhance management efficiency, improve care quality, and generate financial benefits for the organization.

Achieving these outcomes requires a clear digital health strategy, robust governance structures, optimized operational processes, and a qualified workforce capable of leveraging technology to its full potential.

Maturity Models (MMs) have evolved beyond their original use in evaluating software vendors and development processes. Today, they are widely applied as tools for benchmarking, self-assessment, change management, and organizational learning. The adoption and increasing maturity of these models within hospital management contribute directly to improved operational efficiency and profitability.

Furthermore, strict regulatory environments also benefit from the application of MMs, particularly when these models are embedded into organizational processes and supported by HIS. This helps healthcare organizations demonstrate compliance and readiness in areas such as data governance, cybersecurity, and quality management.

Many of these Maturity Models draw inspiration from the Capability Maturity Model (CMM) and Capability Maturity Model Integration (CMMI), frameworks that have influenced dozens of healthcare-specific MMs. Some of these models have been developed by national and supranational health organizations, as well as by leading technology corporations focused on advancing healthcare digital transformation. These models provide structured pathways for improving health IT capabilities and achieving more integrated, patient-centered, and cost-effective care delivery.

### Compliance with Ethical Standards

The authors declare no conflicts of interest. This article does not involve any studies with human participants or animals conducted by the authors. Therefore, formal consent was not required for this type of study.

## Figures and Tables

**Table 1:** Search criteria.

Search Criteria
Maturity Model AND Health
Maturity Model AND Healthcare
Maturity Model AND Hospital
Maturity Model AND eHealth
Maturity Model AND HIS
Maturity Model AND Health Information System

**Table 2:** Networkability maturity components and related factors.

Components	Factors
Strategic alignment	Communication, partnership, strategy governance
IT management	IT governance, IT organization, IT performance, IT scope, IT strategy
Process management	BPM (business process management) alignment, BPM methods, BPM governance people
Organizational project management	OPM governance, OPM assessment, OPM communication, People cooperation
Cooperation management	Collaboration engineering, committee work, cooperation strategy, partner selection
Systems architecture	IT architecture, IT applications, IT integration

**Table 3:** Dimensions across the functional areas.

Functional	Technological	Diffusional	Organizational
F_1_—Goal definition	T_1_—BI architecture	D_1_—Accessing users	O1—BI strategy
F_2_—Measurement	T_2_—Reporting	D_2_—System users	O2—BI budget
F_3_—Gap analysis	T_3_—Interface	D_3_—Process coverage	O3—Organization coverage
F_4_—Data quality	T_4_—User profiling		O4—Key-user capabilities
F_5_—Functional integration	T_5_—Technological integration		O5—User capabilities
	T_6_—Standards		O6—Competence improvement
	T_7_—Data provisioning		O7—Partner/supplier coordination

**Table 4:** Comparative summary.

Model Type	Primary Focus	Typical Dimensions	Example Models
**Process-oriented**	Workflows, culture, process quality	Process areas, structure, IT alignment	CMMI, BPOMM, PMMM
**Technology-focused**	Systems, infrastructure, security	IT systems, EMR adoption, cybersecurity	EMRAM, INFRAM, HCSMAF
**Specialized domain**	Specific health services	Domain-specific practices and processes	PACS MM, TMSMM, UMM
**Data and Analytics**	Data management and use	Data quality, analytics, governance	ICMM, HAAM, HDQM2
**Policy-oriented**	Governance and public health policy	Strategy, legislation, national programs	MMHiAP, GDHI, IMAPS

**Table 5:** Summary of Maturity Models for healthcare.

Process-Oriented Models				
	Model Name	Health Focus	Dimensions/Factors	Author
1	Capability Maturity Model Integration for Services (CMMI)	Health care services	24 process areas	[82]
2	Business Process Orientation Maturity Model (BPOMM)	Process orientation	Seven dimensions subdivided into two parts: the BPO-Components and the BPO-Impacts	[83]
3	Process management Maturity Model (PMMM)	Process management	Culture: strategy; structures; practices; IT	[71]
4	Information Management Maturity Assessment Program (IMMAP)	Information management	Measure performance against the whole of Victorian government IM standards. Assess an organization’s ability to meet information management best practice	[89]
5	Networkability maturity Model (NMM)	Networkability	Strategic alignment, IT management, process management, organizational project management, corporation management, system architecture	[90]
6	General Practice Information Maturity Model (GPIMM)	General practice information	Paper-based, computerized, computerized PHCT, coded, bespoke, paperless	[91]
Technology-Focused Models				
7	HIMSS Analytics Electronic Medical Record Adoption Model (EMRAM)	HIS application	Clinical quality; efficiency; patient safety; analytics; interoperability	[92]
8	Electronic Healthcare Maturity Model (eHMM)	Standardizing, integrating, and optimizing electronic processes	Timeliness of process; data access and accuracy of data; process effort; cost-effectiveness; quality of process results; utility or value to stakeholders. The eHMM proposes a 7-level Maturity Model	[96]
9	IDC Maturity Scapes (IDC-MS)	3rd Platform technologies	Intent; data; technology; people; processes	[98]
10	IDC Mobility Maturity Model (IDC-Mobility)	Mobility	Strategic intent; technology; people; processes	[100]
11	IDC healthcare IT Maturity Model (IDC-HIT)	Integrated Health Information Systems	Improved clinical outcomes; patient safety; operational efficiency; continuity and coordination of care; access and equity; data-driven health governance	[103,104]
12	Healthcare Information Technology Maturity Model (HIT-MM)	Digitization and integration of systems	Clinical quality; patient safety; operational efficiency; data-driven governance; patient engagement; health equity and access	[105]
13	NHS Infrastructure Maturity Model (NIMM)	Evaluating and advancing NHS IT infrastructure	People and organization; technology; security and information governance; alignment and value	[106]
14	PACS Maturity Model (PMM)	PACS	Strategy and policy; organization and processes. Monitoring and control; IT; people and culture	[108]
15	Hospital Information System Maturity Model (HISMM)	Assess and guide the evolution of HIS	Data analysis; strategy; people, electronic medical record; information security; systems and IT infrastructure	[110]
16	Forrester “Meaningful Use” Model	EMR	Access; interoperability; content features; planning and strategy	[112]
17	IGHealthRate™	Information governance	(1) IG structure; (2) strategic alignment; (3) enterprise information management; (4) privacy and security; (5) legal and regulatory;(6) data governance; (7) IT governance; (8) analytics; (9) IG performance; (10) awareness and adherence.	[113]
18	Public Health Information Technology (PHIT)	Information technology	Scale and scope of use PHIT, PHIT quality, PHIT human capital, policy and resources, PHIT community infrastructure	[114]
19	IT Maturity Model for Smart City Services in Emerging Economies (FSCE2)	Smart city services	A conceptual model of smart cities services, IT dimensions and indicators, IT maturity levels Integrated (level 1), analytically managed (level 2, optimized automated (level 3)	[116]
20	Cloud Security Capability Maturity Model (CSCMM)	Cloud security	Domain and maturity level 12 security domains and 4 levels of maturity	[118]
21	Healthcare Cloud Security Maturity Assessment Framework (HCSMAF)	Cloud security	Identity and access management, data privacy and management, risk management, asset management, cryptography and key management, infrastructure and network security, compliance and audit management, incident response management, business continuity management. Initial (level 1): managed (level 2), quantitatively managed (level 3): optimizing (level 4)	[119]
22	Health Information Systems Interoperability Maturity Toolkit (HISAMT)	Information systems interoperability	Nascent, emerging, established, institutionalized and optimized	[123]
23	Digital Health Profile and Maturity Assessment Toolkit (DHPMAT)	Support national health priorities	Level 1: basic, Level 2: controlled, Level 3: standardized, Level 4: optimized, Level 5: innovative	[54]
24	Information Systems for Health Standard Assessment Method (IS4HMM)	Information systems for health	Conceptual framework; tactical framework; strategic framework; concepts, process, services and products; trust-based model; learning framework	[126]
25	Infrastructure Adoption Model (INFRAM)	Align IT capability	Cybersecurity; IT management and performance; adoption; outcomes; sustainability. Each of these dimensions is assessed across a maturity scale (Levels 0–7) to determine how well the infrastructure contributes to overall healthcare goals	[127]
Specialized Domain Models				
26	The Telemedicine Service Maturity Model (TMSMM)	Telemedicine	Man; machine; material; method; money	[128]
27	Continuity of Care Maturity Model (CCMM)	EMR	HIS application	[129]
28	Interoperability Maturity Model (IMM)	Interoperability	Organization, information, technical	[131]
29	Health Information Network Maturity Model (HIN)	Health information exchange	Vision and engagement; governance, policy and legislation; skills and resources; financing; model practice; success metrics; clinical use cases; technology and apps; security and privacy	[133]
30	Healthcare Usability Maturity Model (UMM)	Usability	Focus on users; management; process and infrastructure; resources; education	[158]
31	Hospital Cooperation Maturity Model (HCMM)	Networking/cooperation	Strategic; organizational, information	[138]
32	Health Game Maturity Model (HGMM)	Gamification	Value; process; coverage; types	[139]
33	Maturity Model for Hospital (MMH)	Medical service improvement	Learning and growth; hospital’s process; patients; citizen	[140]
34	High Reliability Health Care Maturity Model (HRHCM)	High reliability health care	Leadership, safety culture; robust process improvement	[142]
35	GRID Interoperability Maturity Model (G-IMM)	Interoperability	Organizational, informational, and technical	[143]
36	Global Goods Maturity Model (GGMM)	Open-source mobile and web application software	Global utility, community support, and software maturity global indicators	[143]
37	The Field Hospital Maturity Model (FHMM)	Field hospitals	Governance, logistics, and care Unconsidered, initial, practiced, managed, and improved	[145]
38	Interoperability Maturity Assessment of a Public Service (IMAPS)	Interoperability	Service delivery, service consumption, service management. Ad hoc (level 1): opportunistic (level 2): essential (level 3): sustainable (level 4): seamless (level 5)	[146]
39	Health Information System Stages of Continuous Improvement Toolkit (SOCI)	System stages of continuous improvement	Emerging/ad hoc, repeatable, defined, managed, and optimized	[147]
40	Community Care Outcomes Maturity Model (C-COMM)	Integration	Digital care coordination; access and equity; patient and caregiver engagement; outcomes and performance measurement; community partnerships and ecosystem integration; digital maturity and infrastructure readiness	[148]
41	Integrated Care Maturity Model (IC-MM)	Infrastructure IT	Managing IS; using BI; using IT; (4) aligning business and informatics; managing change. Five levels of maturity: basic, controlled, standardized, optimized, and innovative	[149]
42	Collaboration Maturity Model (CollabMM)	Coordination	Process integration; governance and roles; supporting tools; measurement and improvement. Similar to other Maturity Models, uses five progressive levels	[150]
Data and Analytics Models				
43	Informatics Capability Maturity Model (ICMM)	eHealth	The dimensions considered are as follows: ICM1. Collection, integration, and management of data in HIS/HER; ICM2. Sharing information in the health district; ICM3. Manage the implementation and change of information and communication technology in health; ICM4. Data quality management and information governance; ICM5. Using healthcare business intelligence to improve population care and health	[136,151]
44	The Healthcare Analytics Adoption Model (HAAM)	Data warehouse and analysis	New data sources; complexity; data literacy; data timeliness	[152]
45	Business Intelligence Maturity Model (BIMM)	Business intelligence	Collaboration; knowledge; trust; institutions; governance	[153]
46	Healthcare Data Quality Maturity Model (HDQM2)	Data quality	Accuracy/correctness; completeness; uniqueness; duplicates	[156]
47	Data Quality Maturity Model (DDMM)	Data quality	Conceptual, reactive, structured, complete, and advanced	[157]
48	Adoption Model for Analytics Maturity (AMAM)	Predictive analytics and governance	The AMAM is an international eight-stage (0–7) model measuring the capabilities that organizations have gained from technology and surrounding processes	[163]
Policy-Oriented Models				
49	The Maturity Model for Health in All Policies (MMHiAP)	Health in all policies at local government	Unrecognized, recognized, considered, implemented, integrated, institutionalized	[159,160]
50	Global Digital Health Index (GDHI)	Global maturity assessment in digital health and benchmark against other countries	Leadership and governance; strategy and investment; legislation, policy, and compliance; workforce; standards and interoperability; infrastructure; services and applications	[161]

## Data Availability

The original contributions presented in this study are included in the article. Further inquiries can be directed to the corresponding author.
